# Ubiquitin Carboxyl-Terminal Hydrolase L1 (UCHL1) Promotes Uterine Serous Cancer Cell Proliferation and Cell Cycle Progression

**DOI:** 10.3390/cancers12010118

**Published:** 2020-01-02

**Authors:** Suet-Ying Kwan, Chi-Lam Au-Yeung, Tsz-Lun Yeung, Angela Rynne-Vidal, Kwong-Kwok Wong, John I. Risinger, Hui-Kuan Lin, Rosemarie E. Schmandt, Melinda S. Yates, Samuel C. Mok, Karen H. Lu

**Affiliations:** 1 Department of Gynecologic Oncology and Reproductive Medicine, The University of Texas MD Anderson Cancer Center, Houston, TX 77030, USA; 2 The University of Texas Graduate School of Biomedical Sciences at Houston, Houston, TX 77030, USA; 3 Department of Obstetrics, Gynecology, and Reproductive Biology, College of Human Medicine, Michigan State University, Grand Rapids, MI 48824, USA; 4 Department of Cancer Biology, Wake Forest School of Medicine, Winston-Salem, NC 27157, USA; 5 Graduate Institute of Basic Medical Science, China Medical University, Taichung 404, Taiwan; 6 Department of Biotechnology, Asia University, Taichung 413, Taiwan

**Keywords:** endometrial cancer, uterine serous carcinoma, UCHL1, cyclin B1, The Cancer Genome Atlas

## Abstract

Uterine serous carcinoma (USC) is the most aggressive form of endometrial cancer, with poor survival rates and high recurrence risk. Therefore, the purpose of this study was to identify therapeutic targets that could aid in the management of USC. By analyzing endometrial cancer samples from The Cancer Genome Atlas (TCGA), we found Ubiquitin Carboxyl-Terminal Hydrolase L1 (UCHL1) to be highly expressed in USC and to correlate with poorer overall survival. UCHL1 silencing reduced cell proliferation in vitro and in vivo, cyclin B1 protein levels and cell cycle progression. Further studies showed that UCHL1 interacts with cyclin B1 and increases cyclin B1 protein stability by deubiquitination. Treatment of USC-bearing mice with the UCHL1-specific inhibitor reduced tumor growth and improved overall survival. Our findings suggest that cyclin B1 is a novel target of UCHL1 and targeting UCHL1 is a potential therapeutic strategy for USC.

## 1. Introduction

Endometrial cancer is the most common gynecologic malignancy in the United States [1] and is categorized into two broad subtypes based on histopathology [2]. Type I tumors are grade 1–2 endometrial endometrioid carcinomas (EEC) with a favorable prognosis, while type II malignancies have a poorer prognosis and include grade 3 endometrioid carcinoma, uterine serous carcinoma (USC) and clear cell carcinoma.

While the majority of tumors are EEC, USC is the most common non-endometrioid subtype and the most aggressive, accounting for approximately 10% of all endometrial cancers but 40% of deaths [3]. Poor survival outcome is partly due to the high percentage of cases diagnosed at stage III and IV (41.3% as opposed to 13.9% of EEC [4]). Unlike type I tumors, risk of metastasis and recurrence cannot be predicted from tumor size, grade, and depth of myometrial invasion [3,5]. Disease stage, presence of extra-uterine disease and suboptimal cytoreduction are risk factors for overall survival [6,7], but both early- and late-stage USC behave aggressively, with a tendency toward lymphovascular invasion, intraperitoneal disease and extra-abdominal spread [3]. Therefore, if any USC component is present in the primary tumor, patients are considered high risk for treatment purposes. Complete surgical staging is performed, comprising total hysterectomy, bilateral salpingo-oophopherectomy and lymph node dissection [8], followed by carboplatin and paclitaxel [9,10].

The 5-year disease-specific survival for USC is approximately 74% and 33% for early-stage and late-stage patients [11], compared to 89% and 77% for low-grade and grade 3 EEC [12]. In light of poor patient survival and high recurrence rates, the development of targeted therapies specific to USC pathway aberrations would aid in its management. However, conducting studies that focus solely on this rare subtype have been a constant challenge, making it difficult to determine optimal treatment strategies [9].

In this study, we first utilized the data from The Cancer Genome Atlas (TCGA) to determine which genetic changes were responsible for the increased aggressiveness and poorer survival outcomes in USC. Focusing on Ubiquitin Carboxyl-Terminal Hydrolase L1 (UCHL1), a deubiquitinating enzyme that has a putative oncogenic role in various cancer types [13,14,15,16,17,18], we sought to elucidate the molecular mechanism by which UCHL1 promotes tumor progression in USC, and evaluated the effect of targeting UCHL1 on tumor cell proliferation in vivo.

## 2. Results

### 2.1. UCHL1 Is Upregulated in USC and Is Significantly Correlated with Poorer Overall Survival

To identify genes contributing to the increased aggressiveness of USC, we used the TCGA data set to find genes that are upregulated in USC and associated with poorer overall survival. To identify upregulated genes, we compared the RNA expression profiles of USC to low grade EEC and normal adjacent endometrial tissue. Grade 3 EEC was not included in the EEC group, as we observed an overlap between the RNA expression profiles of grade 3 EEC and USC based on principal component analysis.

**Figure 1:**
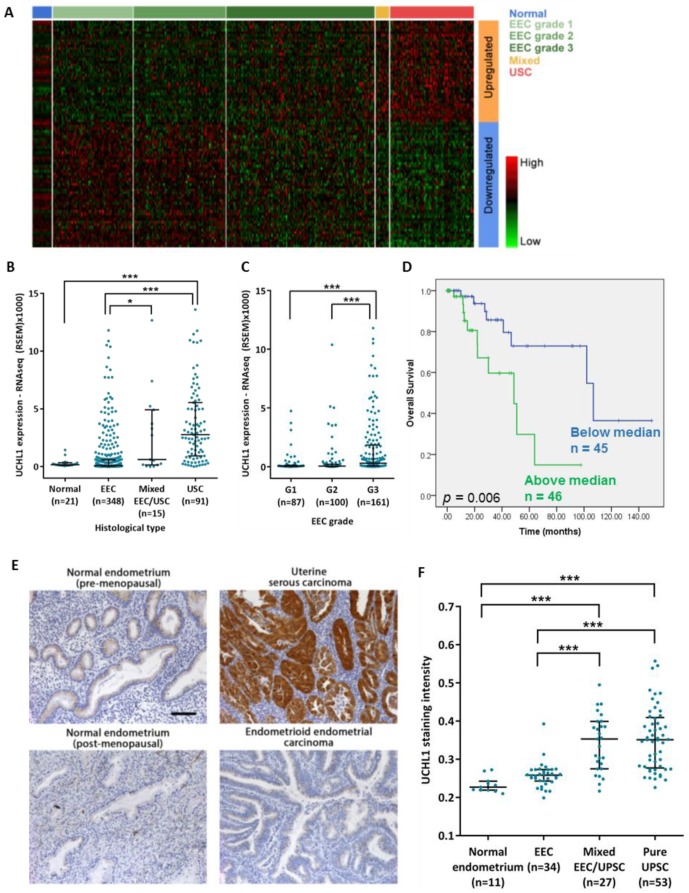
UCHL1 is upregulated in USC and correlates with poorer overall survival. (**A**–**D**) Analysis of the endometrial cancer TCGA data set. (**A**) Heat map of the genes differentially expressed between USC and low grade EEC, with a significant association with overall survival in USC patients. (**B**) UCHL1 RNA expression across histological subtypes of endometrial cancer (Normal, *n* = 21; EEC, *n* = 348; Mixed EEC/USC, *n* = 15; USC, *n* = 91). (**C**) UCHL1 RNA expression across EEC grades (G1, *n* = 87; G2, *n* = 100; G3, *n* = 161). Statistical significance for (**B**,**C**) was determined by the Kruskal-Wallis test. Error bars represent median with interquartile range. * *p* < 0.05, *** *p* < 0.001. (**D**) Kaplan-Meier analysis of UCHL1 expression and overall survival in USC patients (*n* = 91). Statistical significance was determined by the log-rank test (*p* = 0.006). (**E**,**F**) Validation of UCHL1 upregulation in an independent cohort of paraffin-embedded tumor samples. (**E**) Representative slides for UCHL1 staining in various gynecological tissues. Scale bar, 100 µm. (**F**) UCHL1 staining intensity across tissue types (Normal endometrium, *n* = 11; EEC, *n* = 34; Mixed EEC/USC, *n* = 27; Pure USC, *n* = 53). Statistical significance was determined by the Kruskal-Wallis test. Error bars represent median with interquartile range. *** *p* < 0.001.

Significance Analysis of Microarrays (SAM) [19] was used to compare between low-grade EEC and USC, and also between the adjacent normal samples and USC. 1968 genes were differentially expressed in both comparisons (*q* < 0.05, fold change ≥ 2). Of these, 85 genes were significantly associated with overall survival of patients with pure USC (*p* < 0.05) (Figure 1A). Of particular interest were the 19 genes that were significantly higher in USC compared to both normal tissue and low-grade EEC, and for which high expression was associated with poorer overall survival (Table S1).

Of these, we chose to study UCHL1 further, due to it being the most highly expressed in the USC group. UCHL1 exhibited over a 40-fold increase in expression from low-grade EEC to USC (Figure 1B). In addition, expression in grade 3 EEC tumors was significantly higher than low-grade EEC (Figure 1C). Expression was not significantly different across stages of USC, suggesting that its upregulation may be an early event. Finally, high expression correlated with poorer overall survival of USC patients by both Kaplan-Meier analysis (Figure 1D) and multivariate analysis (Table S2). In contrast, UCHL1 expression did not predict prognosis in patients with grade 3 EEC. USC patients grouped below and above median UCHL1 expression were similarly treated. Amongst patients with information on surgical approach, 15/42 (36%) and 27/42 (64%) of those with expression below the median, and 14/45 (31%) and 31/45 (69%) of those with expression above the median, underwent minimally invasive surgery or open surgery respectively. Amongst patients with information on drug treatments, 22/22 (100%) patients with expression below the median and 20/22 (91%) patients with expression above the median also received chemotherapy.

To validate our findings, immunohistochemical staining was performed in an independent patient cohort (demographics in Table S3). UCHL1 staining intensity was significantly higher in pure and mixed USC than in normal tissue and ECC (Figure 1E,F), and was not significantly associated with age, ethnicity, pure vs. mixed histology or stage in the USC group. The majority of USC tumors exhibited diffuse cytoplasmic staining and occasional nuclear staining. Furthermore, compared to primary tumors from the same patient, UCHL1 expression was increased in 5 out of 6 omental metastases and 4 out of 4 lymph node metastases (Figure S1A,B).

There was no significant association between UCHL1 expression and survival when analyzing early stage patients alone or all patients together in our validation cohort. However, in the 30 late stage patients with no evidence of disease after completion of treatment, high UCHL1 expression was significantly associated with poorer disease-free survival by univariate and multivariate analysis, and overall survival by multivariate analysis (Figure S1C,D and Table S4).

### 2.2. UCHL1 Silencing and Inhibition Suppresses USC Growth In Vitro and In Vivo

**Figure 2:**
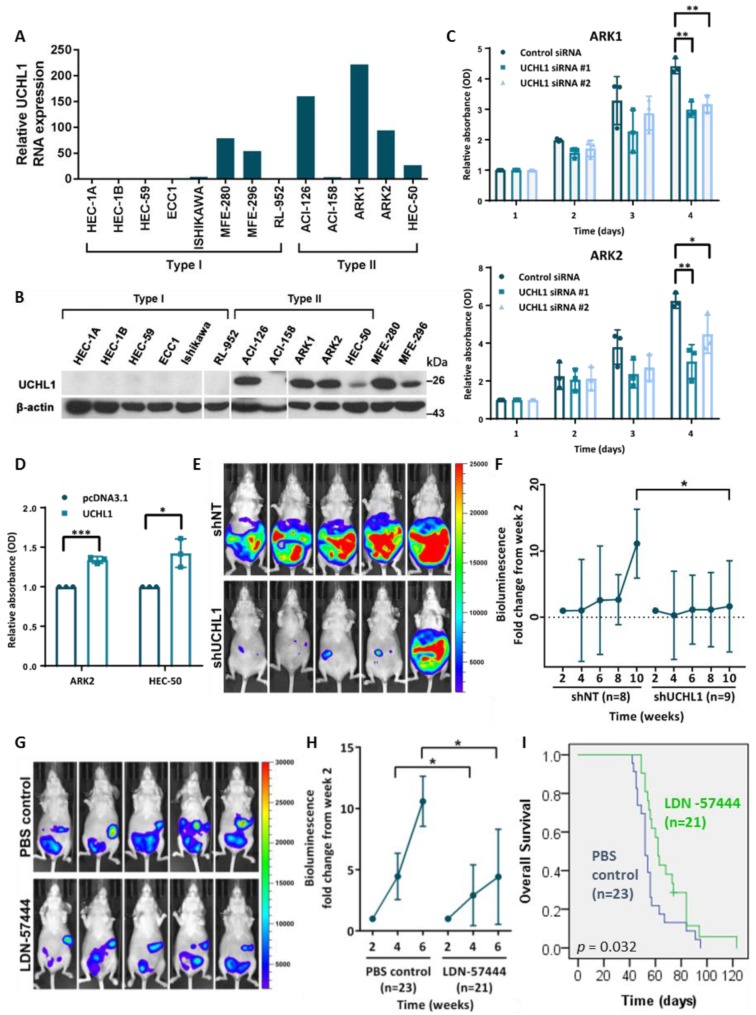
UCHL1 silencing reduces USC cell proliferation in vitro. (**A**) qRT-PCR quantification of UCHL1 RNA expression in endometrial cancer cell lines (Type I, *n* = 8; Type II, *n* = 5). (**B**) Western blot analysis of UCHL1 protein expression in endometrial cancer cell lines (Type I, *n* = 8; Type II, *n* = 5). (**C**,**D**) Cell proliferation of ARK1 and ARK2 cells after UCHL1 knockdown (**C**), and of ARK2 and HEC-50 cells stably overexpressing UCHL1 (**D**), as measured by the MTT assay. Statistical significance was determined by the *T*-Test (two-tailed, equal variance). Error bars represent mean ± SD (3 biological replicates). * *p* < 0.05, ** *p* < 0.01, *** *p* < 0.001. (**E**,**F**) Luciferase-labelled ARK1 cells transduced with doxycycline-inducible control shRNA (*n* = 8) or anti-UCHL1 shRNA (*n* = 9) were injected intraperitoneally into nude mice; the mice were then switched to a doxycycline diet the following day. (**E**) Representative images of in vivo bioluminescence imaging at week 10. (**F**) Fold change increase in in vivo bioluminescence of intraperitoneal tumors. Statistical significance was determined by the Mann-Whitney test. Error bars represent mean ± SD. * *p* < 0.05. (**G**–**I**) Luciferase-labelled ARK1 cells were injected intraperitoneally into nude mice; after 2 weeks, mice were treated thrice weekly by intraperitoneal injection of LDN-57444 (*n* = 21) or PBS control (*n* = 23). (**G**) Representative images of in vivo bioluminescence imaging at week 6. (**H**) Fold change increase in in vivo bioluminescence of intraperitoneal tumors. Statistical significance was determined by the Mann-Whitney test. Error bars represent mean ± SD. * *p* < 0.05. (**I**) Kaplan-Meier survival curves of the LDN-57444 treatment group (*n* = 21) and control group (*n* = 23). Statistical significance was determined by the log-rank test (*p* = 0.032). The uncropped blots and molecular weight markers are shown in Supplementary Materials.

UCHL1 RNA and protein expression was undetectable in cell lines derived from type I endometrial tumors except for MFE-280 and MFE-296, but detected in all type II cell lines except ACI-158 (Figure 2A,B). All type II cell lines were *TP53*-mutants (Figure S2A,B), reflecting frequent *TP53* mutation in USC tumors.

The effect of UCHL1 silencing on apoptosis, migration and proliferation was determined using ARK1 and ARK2 cells. Notable knockdown was achieved by 72 h after siRNA transfection (Figure S2C). While there was no significant change in apoptosis or cell migration following UCHL1 silencing (Figure S2D,E), we observed a significant reduction in cell proliferation in ARK1 and ARK2 cells (Figure 2C) but not the UCHL1-negative ACI-158 cells (Figure S2F). Additionally, UCHL1 overexpression increased cell proliferation of ARK2 and HEC-50 cells (Figure 2D).

To determine whether UCHL1 also promotes proliferation in vivo, we established tumors using ARK1 transduced with doxycycline-inducible control shRNA (ARK1-luc-dox-shNT) or anti-UCHL1 shRNA (ARK1-luc-dox-shUCHL1); successful induction by doxycycline was previously confirmed in vitro (Figure S2G). By week ten, mice with ARK1-luc-dox-shUCHL1 tumors had significantly lower fold change increase in bioluminescence from week 2 (Figure 2E,F), and showed a trend towards improved overall survival (Figure S2H).

The effect of the UCHL1 inhibitor LDN-57444 [20] on USC growth was also examined. Treatment of UCHL1-positive cells ARK1, ARK2 and HEC-50 inhibited cell proliferation at the micromolar level (Figure S2I). In addition, treatment of nude mice with LDN-57444 reduced the growth of ARK1 tumors compared to control treatment (Figure 2G,H) and improved overall survival (Figure 2I); there was no significant decrease in body weight, suggesting a lack of systemic toxicity (Figure S2J). Overall, this suggests that UCHL1 inhibition has an anti-proliferative effect both in vitro and in vivo, and that UCHL1 is a viable therapeutic target.

### 2.3. UCHL1 Upregulates Cyclin B1 Protein Expression

To identify potential downstream targets of UCHL1 that mediate its effects in USC progression, we identified genes whose protein expression levels correlated with UCHL1 expression in the TCGA data set. As UCHL1 is a deubiquitinating enzyme, of particular interest were the genes affected by UCHL1 expression at the protein level but not at the RNA level. Of 166 probes, 17 proteins were significantly correlated at the protein level. Of these, 6 proteins (cyclin B1, p21, GSK3α, GSK3β, MSH2, RAD50) were positively correlated with UCHL1 expression at the protein level but not at the RNA level, suggesting that their protein stability may be increased by the deubiquitinating activity of UCHL1.

**Figure 3:**
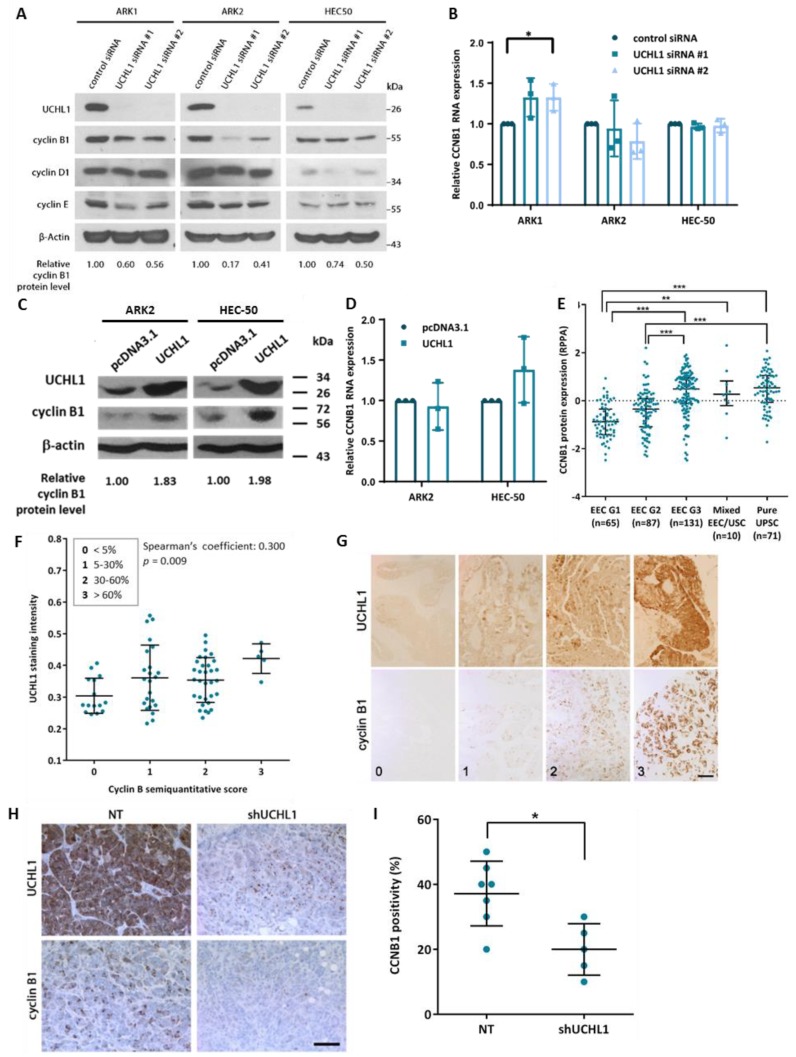
Cyclin B1 protein is upregulated by UCHL1. (**A**) Expression of cyclin proteins four days after siRNA transfection of ARK1, ARK2 and HEC-50 cells. Representative experiment (3 biological replicates). (**B**) qRT-PCR quantification of cyclin B1 RNA expression four days after siRNA-mediated UCHL1 silencing. Statistical significance was determined by the *T*-Test (two-tailed, equal variance). Error bars represent mean ± SD (3 biological replicates). * *p* < 0.05. (**C**) Cyclin B1 protein expressions in ARK2 and HEC-50 cells stably overexpressing UCHL1. Representative experiment (3 biological replicates). (**D**) qRT-PCR quantification of cyclin B1 RNA expression in ARK2 and HEC-50 cells stably overexpressing UCHL1. Error bars represent mean ± SD (3 biological replicates). (**E**) Cyclin B1 protein levels across histological subtypes of endometrial cancer (TCGA data set). Statistical significance was determined by the Kruskal-Wallis test. Error bars represent median with interquartile range. ** *p* < 0.01, *** *p* < 0.001. (**F**) Correlation between cyclin B1 positivity and UCHL1 staining intensity in the USC cohort. Statistical significance was determined by the Spearman’s test. Error bars represent mean ± SD. (**G**) Representative slides of samples stained for both cyclin B1 and UCHL1 with increasing semiquantitative score. Scale bar, 100 µm. (**H**) Immunohistochemical staining of cyclin B1 and UCHL1 in tumors established by intraperitoneal injection of nude mice with ARK1 cells transduced with doxycycline-inducible control shRNA (*n* = 7) or anti-UCHL1 shRNA (*n* = 5). Representative slides. Scale bar, 100 µm. (**I**) Percentage positivity of cyclin B1 in tumor cells at time of sacrifice. Statistical significance was determined by the Mann-Whitney test. Error bars represent mean ± SD. * *p* < 0.05. The uncropped blots and molecular weight markers are shown in Supplementary Materials.

Following siRNA-mediated silencing of UCHL1, we could not observe a consistent decrease in the cell-cycle related proteins p21, GSK3α or GSK3β (Figure S3). We also did not see a consistent change in p53, p27 or β-catenin protein levels, which were previously reported cell-cycle related targets of UCHL1 [21,22,23]. In contrast, UCHL1 silencing led to a reduction in cyclin B1 levels in ARK1, ARK2 and HEC-50 cells (Figure 3A). The other major cyclins involved in the cell cycle, cyclin D1 and cyclin E1, were not consistently decreased by UCHL1 silencing. Examination of RPPA and RNAseq data from just the pure USC tumors in the TCGA data set also confirmed that while there was a significant positive correlation between UCHL1 RNA expression and cyclin B1 protein, CDK1 showed only a trend towards positive correlation with UCHL1, and there was no significant correlation for cyclin D1 or cyclin E1 protein (Figure S4). In addition, the decrease in cyclin B1 protein was not accompanied by reduced RNA expression (Figure 3B). Conversely, overexpression of UCHL1 increased cyclin B1 protein levels (Figure 3C) without significantly increasing RNA expression (Figure 3D), suggesting UCHL1 affects cyclin B1 protein stability and not RNA transcription.

Cyclin B1 protein expression was significantly higher in USC and grade 3 EEC than in low-grade EEC in the TCGA data set (Figure 3E). In addition, the percentage of tumor cells positive for cyclin B1 was positively correlated with UCHL1 staining intensity in our validation cohort of USC samples (Figure 3F,G). Finally, cyclin B1 positivity was reduced in the tumors established from the UCHL1-silenced ARK1 tumors in our in vivo study compared to control tumors (Figure 3H,I), suggesting that UCHL1 regulates cyclin B1 protein expression both in vitro and in vivo.

### 2.4. UCHL1 Interacts with Cyclin B1

**Figure 4:**
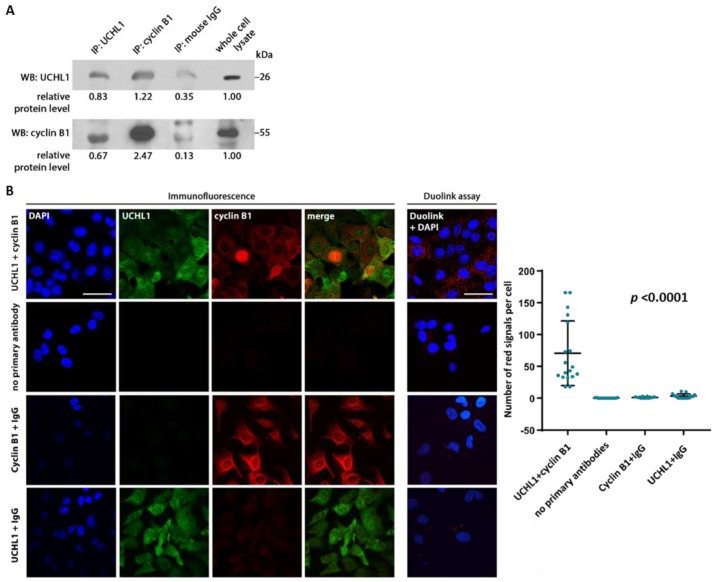
UCHL1 and cyclin B1 colocalize and interact in vitro. (**A**) Co-immunoprecipitation of ARK1 protein lysate was performed using antibodies against UCHL1 and cyclin B1, with normal mouse IgG as a control. (**B**) Immunofluorescence staining and the Duolink proximity ligation assay was performed on ARK1 cells. Following the Duolink assay, red fluorescent dots indicate presence of protein-protein interaction. As controls, ARK1 cells were also stained without primary antibody, with anti-UCHL1 antibody plus control IgG, and with anti-cyclin B1 antibody plus control IgG. Representative experiment (3 biological replicates). Scale bar, 50 µm. Results in the dot plot show the number of red signals per cell in three independent experiments. Statistical significance was determined by the Kruskal-Wallis test. Error bars represent mean ± SD (*p* < 0.0001). The uncropped blots and molecular weight markers are shown in Supplementary Materials.

Cyclin B1 is a positive regulator of cell cycle progression. We first determined whether UCHL1 interacts with cyclin B1. Co-immunoprecipitation indicated interaction between the two proteins (Figure 4A), which were also shown to colocalize through immunofluorescence imaging (Figure 4B). This was further supported by the Duolink proximity ligation assay, with interaction observed in ARK1 and ARK2 cells but not UCHL1-negative HEC-1A cells (Figure 4B and Figure S5A). UCHL1 silencing abrogated the interaction observed (Figure S5B).

**Figure 5:**
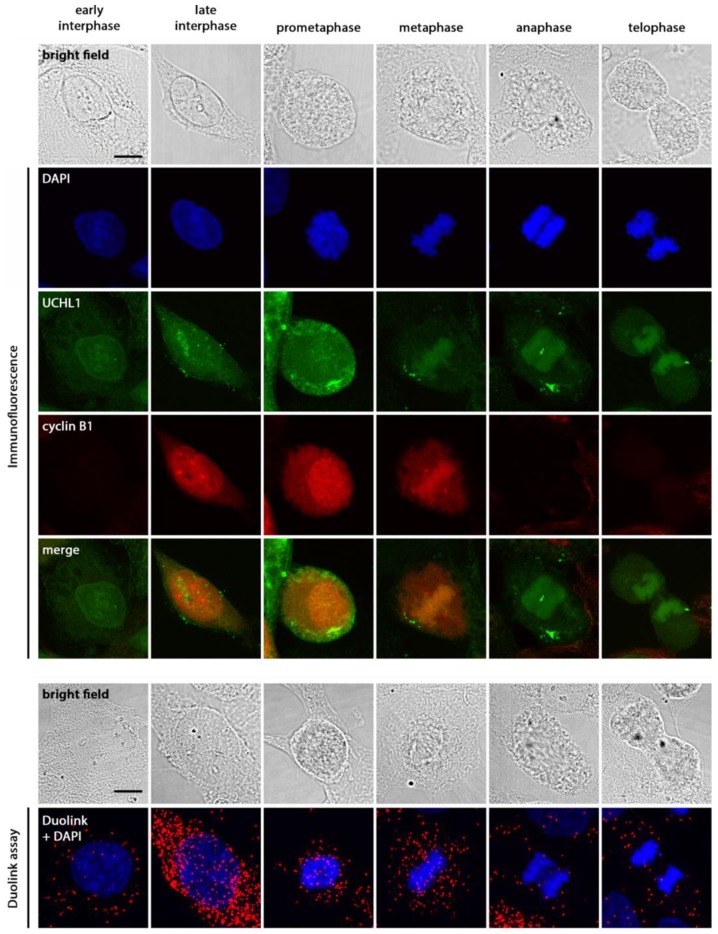
UCHL1 and cyclin B1 interact during interphase and mitosis. To observe the interaction of UCHL1 and cyclin B1 through mitosis, ARK1 cells were synchronized in early S phase by double thymidine block (16 h of 2 mM thymidine treatment in normal culture medium, followed by 10 h release in culture medium, and a second thymidine block of 14 h). Cells were then collected 10 h after final release to enrich for mitotic cells. Under normal conditions, cyclin B1 protein accumulates from S phase to G2/M and peaks at the onset of mitosis [24]. Representative experiment (3 biological replicates). Scale bar, 10 µm.

In ARK1 cells, the majority of UCHL1-cyclin B1 interaction observed was cytoplasmic; however, when cyclin B1 entered the nuclear space during late interphase (Figure 5), interaction between the two proteins was also observed, which persisted through mitosis until cyclin B1 degradation. Overall, these results suggest that UCHL1 and cyclin B1 are in close proximity and interact in all phases of the cell cycle where cyclin B1 is present.

### 2.5. UCHL1 Increases Protein Stability of Cyclin B1 and Promotes Cell Cycle Progression

**Figure 6:**
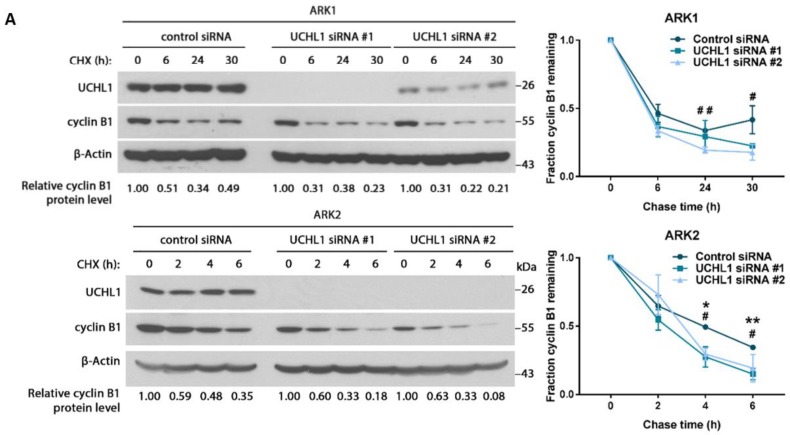

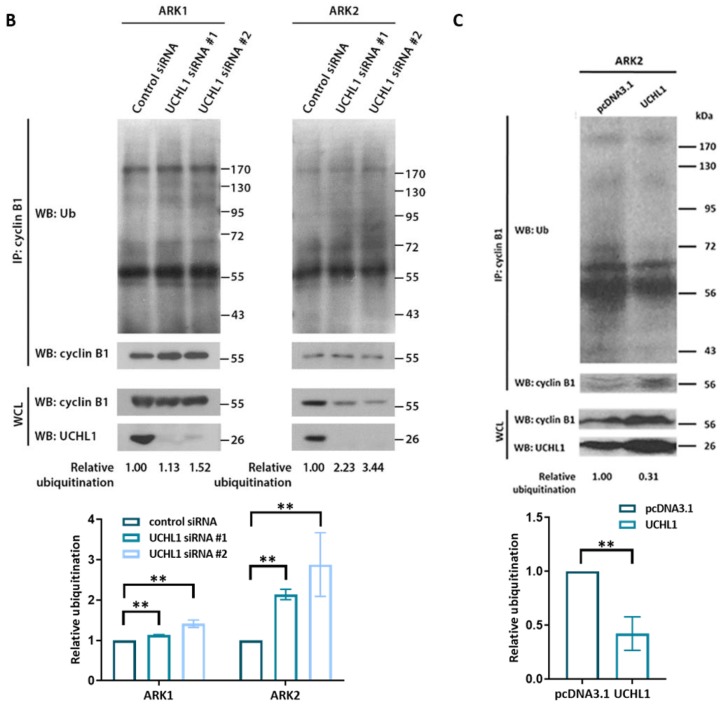
UCHL1 modulates cyclin B1 protein expression by deubiquitination. (**A**) Cycloheximide (CHX) chase assay of cyclin B1 was performed in ARK1 and ARK2 cells 4 days after transfection with UCHL1 siRNA. Protein lysate was collected at the indicated time points after the addition of CHX. Representative experiment (3 biological replicates). Quantitative results of multiple experiments is shown on the right. Statistical significance was determined by the *T*-Test (two-tailed, equal variance). Error bars represent mean ± SD (3 biological replicates). For UCHL1 siRNA #1: * *p* < 0.05, ** *p* < 0.01; for UCHL1 siRNA #2: # *p* < 0.05, ## *p* < 0.01. (**B**,**C**) Immunoprecipitation of cyclin B1 was performed on protein lysate from ARK1 and ARK2 cells 4 days after siRNA transfection (**B**), and on protein lysate from ARK2 cells stably overexpressing UCHL1 (**C**). Cells were pretreated with MG132 before protein extraction. Representative experiment (3 biological replicates). Whole ubiquitin levels were measured to include K48-ubiquitination, K11-ubiquitination or multiple-monoubiquitination. Ubiquitin staining was quantified with ImageJ and normalized to cyclin B1 staining following immunoprecipitation (IP: cyclin B1, WB: cyclin B1). Degree of ubiquitination is shown relative to cells transfected with control siRNA or pcDNA3.1 accordingly. IP = immunoprecipitation; WCL = whole cell lysate; WB = western blot. Quantitative results of multiple experiments is shown at the bottom. Error bars represent mean ± SD (3 biological replicates). ** *p* < 0.01. The uncropped blots and molecular weight markers are shown in Supplementary Materials.

Under normal conditions, cyclin B1 is rapidly degraded at the anaphase-metaphase transition due to ubiquitination by the anaphase-promoting complex [24]. Since UCHL1 affects the protein stability of target proteins in other cancer types by deubiquitination [22,23,25], we sought to determine whether UCHL1 directly affected the protein stability of cyclin B1. The degradation rate of cyclin B1 protein was increased (Figure 6A) and total ubiquitination levels of cyclin B1 increased (Figure 6B) after UCHL1 silencing. Conversely, overexpression of UCHL1 decreased total ubiquitination levels of cyclin B1 (Figure 6C), suggesting that UCHL1 stabilizes protein levels of cyclin B1 by reducing its ubiquitination and impairing degradation.

Since cyclin B1 is essential for cells to enter mitosis, we sought to determine the effect of UCHL1 on cell cycle progression by G1 synchronization and cell cycle analysis. UCHL1-silenced cells progressed slower through the cell cycle compared to control cells (Figure S6A). In particular, the percentage of control cells in G2/M increased 15.9% from 0 h to 24 h, whereas cells transduced with shRNA 1 and 2 increased 7.6% (*p* < 0.05) and 8.3% (*p* = 0.066) respectively, suggesting that UCHL1 promotes cell cycle progression. In addition, UCHL1 overexpression in HEC-50 cells led to faster progression through the cell cycle (Figure S6B), with the percentage of cells in G2/M increasing 7.8% from 0 h to 6 h, compared to 3.1% in cells transfected with control plasmid (*p* < 0.05). Overall, our results suggest that UCHL1 promotes mitotic entry through stabilization of cyclin B1 protein.

As overexpression of cyclin B1 has been reported to contribute to development of aneuploidy, we also examined whether UCHL1 and cyclin B1 were associated with aneuploidy status in endometrial cancer. When analysing all endometrial tumors in the TCGA data set, a positive correlation with aneuploidy score was observed for both UCHL1 RNA expression and cyclin B1 protein levels (Figure S6C).

## 3. Discussion

UCHL1 loss [22,26,27,28] and overexpression [13,14,15,16,17,18] has been reported in numerous cancer types. Subsequently, there are conflicting reports that UCHL1 is a tumor suppressor [22,27,28] or oncogene [15,29,30]. Such discrepancy between studies may be explained by the discovery of various distinct downstream signaling pathways of UCHL1 in different tumor types, indicating that its net effect is highly context-specific.

While differential expression of UCHL1 between USC and EEC has been briefly reported [31], the role of UCHL1 in USC has not been explored in depth. One study reported poor prognosis of high UCHL1 expression in endometrial cancer patients {Nakao, 2018 #10033}. However, over 90% of samples were of EEC histology and mostly lower grade, and 80% of all samples analysed for UCHL1 protein expression were classified into the low expression group based on protein expression. Our data also shows that UCHL1 is only minimally expressed in normal endometrial tissue and low-grade EEC, but exhibits marked upregulation in high-grade EEC and USC, suggesting that its expression is an indicator of tumor aggressiveness in endometrial cancer. Furthermore, though sample size was small, UCHL1 expression was higher in omental and lymph node metastases compared to primary tumors from the same patient, suggesting that UCHL1 may contribute to the metastatic process. However, it remains to be seen whether UCHL1 is upregulated when tumor cells reach the omental site, or if tumor cells with higher expression in the primary tumor have a survival advantage when reaching the omental site.

High UCHL1 expression was associated with poorer overall survival of patients with USC in the TCGA data set by both univariate analysis and multivariate analysis. In our independent cohort, we did not find an association between UCHL1 expression and survival when analyzing all patients together. However, in the 30 late stage USC patients (pure or mixed) with no evidence of disease after completion of treatment, high UCHL1 expression was significantly associated with poorer disease-free survival by univariate and multivariate analysis, and overall survival by multivariate analysis. UCHL1 may therefore affect the clinical outcome of late-stage USC patients rather than early-stage patients; however, the lack of significance when analyzing early stage patients or all patients may be due insufficient sample size. While USC should be studied as a separate entity from EEC tumors, this highlights the difficulty in doing so with samples from a single institution. Further validation of UCHL1 upregulation would be strengthened by the use of specimens from multiple institutions.

UCHL1 silencing led to a decrease in cell proliferation both in vitro and *in vivo*, while UCHL1 inhibition reduced tumor growth and improved survival in vivo. However, the mechanism through which UCHL1 contributes to tumorigenesis has not been fully elucidated. As a deubiquitinating enzyme from the family of ubiquitin carboxyl-terminal hydrolases [21], UCHL1 has been shown to affect the stability of various cell-cycle-related proteins, including p27 [21], p53 [22], MDM2 [22], and β-catenin [23]. We did not find a correlation between UCHL1 mRNA expression and protein expression of these genes. Instead, we demonstrated for the first time a significant correlation between UCHL1 and cyclin B protein expression. UCHL1 silencing by siRNA reduced cyclin B1 protein levels and protein half-life in vitro; the percentage of cells positive for cyclin B1 was also lower in UCHL1-silenced tumors. This decrease in protein stability was due to an increase in ubiquitination, subsequently slowing the progression of ARK1 cells through the cell cycle.

Our proximity ligation assay indicated that UCHL1 interacts with cyclin B1 during both interphase and mitosis, regardless of cellular location, and is therefore able to impair the degradation of cyclin B1 by the APC at the metaphase-anaphase transition. The balance between ubiquitination by the APC and deubiquitination by UCHL1 could determine the degree and rate of cyclin B1 degradation, and also affect the basal level of cyclin B1 observed in cells as they exit mitosis and enter interphase.

Cyclin B1 protein levels were previously reported to be associated with increasing grade and stage of type I endometrial cancer, as well as poorer cancer-specific survival by univariate analysis [32]. In addition, pathway analysis and clustering of the endometrial tumors in the TCGA data set revealed that dysregulation of mitotic processes is a frequent occurrence in USC, including increase in cyclin B1, CDK1 and cyclin E1 protein expression [33].

Cyclin B1 overexpression shortens cell cycle length [34] and allows tumor cells to bypass checkpoint control [35], leading to uncontrolled proliferation. Furthermore, even a slight retardation of cyclin B1 degradation has been shown to induce reactivation of the spindle checkpoint [36,37], leading to chromosomal instability and generation of polypoid cells, especially in cells with loss of functional p53 [38]. As we did not observe a significant difference in UCHL1 expression between stages of USC, UCHL1 may be overexpressed early on in tumor progression; therefore, its effect on cyclin B1 degradation may contribute to the frequent observation of aneuploidy in USC, especially since the majority of tumors are *TP53*-mutant. Further studies are needed to establish the possible role of UCHL1 in genetic instability.

## 4. Materials and Methods

### 4.1. TCGA Data Sets

Data was directly downloaded from the TCGA Data Portal: http://cancergenome.nih.gov/. Clinical data was downloaded on May 2013. Patients with stage IIA were converted to I in accordance with the 2009 revisions to the surgical staging system for endometrial carcinoma by the International Federation of Gynecology and Obstetrics (FIGO) [39]. Overall survival duration was inferred from “days to death” since initial diagnosis for deceased patients; overall survival duration for censored patients was derived from “days to last known alive” and “days to last follow-up”. Survival data was updated with v1.7 and v2.0 follow-up information as released by the TCGA Data Portal. Level 3 RNAseqV2 data was downloaded on March 2013 and included the global expression profiles of 21 normal, 87 EEC grade 1, 100 EEC grade 2, 161 EEC grade 3, 15 mixed EEC/USC and 91 USC samples. Normalized RSEM data was used for principal component analysis (PCA), while raw counts were used for Significance Analysis of Microarrays (SAM). For the screening of proteins (cyclin B1, CDK1, cyclin D1 and cyclin E1) significantly correlated with UCHL1, level 3 RPPA data was downloaded on November 2019 and included 283 EEC samples, 10 mixed USC/EEC samples, and 71 pure USC samples for which concurrent clinical and RNAseqV2 data were available. Aneuploidy scores for endometrial tumors were downloaded from cBioportal (www.cBioportal.org), under the PanCancer Atlas data set.

### 4.2. Analysis of TCGA Data

#### 4.2.1. Principal Component Analysis

Level 3 normalized RSEM data for normal, EEC, mixed and pure UPSC samples was used for PCA analysis. The signal-to-noise (SNR) ratio of each gene was calculated by dividing the mean expression value across patients over its standard deviation. The data was then filtered to remove genes with a log10 SNR 2 standard deviations below the mean. The 500 genes with the highest variance were selected for analysis; values were log2 transformed and mean-centered before analysis using the “prcomp” function in R.

#### 4.2.2. Significance Analysis of Microarrays (SAM)

The SAM method for sequencing data (version 4.0; Stanford University Labs) was used to identify differentially expressed (DE) genes between low-grade EEC and pure UPSC. Raw counts from the level 3 data for RNAseqV2 were filtered to remove genes with 10 or less reads across all samples, or genes with an average of 0.5 or less across all samples. The remaining 19093 genes were then converted to integers for analysis. For each comparison, two-class (unpaired) analysis was used, and the number of permutations was set to 100. *q*-values were obtained for each gene. Genes were considered DE if *q* < 0.05 and the SAM calculated fold change ≥ 2.

#### 4.2.3. Automated Log-Rank Test of Genes in the RNA Sequencing Data Set

A two-sample log-rank test for overall survival was calculated for every significant gene identified from the SAM assessment using level 3 normalized RSEM data for all 91 pure UPSC samples (57 initial samples plus an additional 34). Vital status and overall survival time were extracted from clinical data as previously described. For each gene, patients were assigned to one of two groups: below or above the median expression value. A loop in R statistical software (version 2.15.0, R Foundation for Statistical Computing) was used to perform the “survdiff” function from the package “survival” on each gene. The computed *p*-values were extracted for each gene. Hazard ratio was calculated for each gene (above median/below median) by HR = (O_1_/E_1_)/(O_2_/E_2_), where O_1_ and O_2_ are the observed number of deaths in the groups below and above the median respectively, and E_1_ and E_2_ are the expected number of deaths in the groups below and above the median respectively.

#### 4.2.4. Spearman’s Test of Correlation between UCHL1 RNA Expression and RPPA Data

Of the pure UPSC samples within the RNA sequencing dataset, 22 also had RPPA data available (3 of which were mixed EEC/UPSC). Using these 22 samples, we performed Spearman’s test of correlation to determine the correlation between UCHL1 RNA expression and each of the 166 protein probes. For probes with *p* < 0.05, Spearman’s test was also performed with their RNA expression levels against UCHL1 RNA expression levels.

### 4.3. Clinical Specimens

Formalin-fixed, paraffin-embedded sections of USC, EEC and normal tissue samples were obtained from the MD Anderson Gynecologic Cancer Translational Research Tissue Bank. Normal premenopausal uterine samples were from individuals without cancer, while normal postmenopausal uterine samples were from tissue adjacent to tumor. Patient samples and clinical data were collected under protocol approved by the MD Anderson Institutional Review Board (IRB) in accordance with U. S. Common Rule with written informed consent from patients (The IRB code is LAB02-188 and the activation date is 6 August 2017). Overall survival duration was calculated from date of initial diagnosis to death, or to last known alive date for censored patients. Disease-free survival was calculated from date of treatment end (surgery or adjuvant therapy) to date of recurrence, or to last known disease-free date for censored patients.

### 4.4. Cell Culture

Human endometrial carcinoma cell lines ECC1, HEC-1A, HEC-1B, HEC-50, HEC-59, Ishikawa, MFE-296, MFE-280 and RL-952 (ATCC) were cultured in RPMI-1640 with 2 nM L-glutamine (Invitrogen, Carlsbad, CA, USA) and 10% fetal bovine serum. Human USC cell lines ARK1 and ARK2 were a kind gift from Dr. Alessandro D. Santin (Yale Cancer Center, New Haven, CT, USA), and USC cell lines ACI-126 and ACI-158 were a kind gift from Dr. John I. Risinger (Michigan State University, East Lansing, MI, USA). All four cell lines were cultured in DMEM with 1.0 g/L glucose, L-glutamine, sodium pyruvate (Corning Cellgro, Corning, NY, USA) and 10% fetal bovine serum. All cells were maintained at 37 °C in 5% CO_2_. Use of the cell lines in experiments were within the first 10 passages. All cells were tested negative for mycoplasma contamination using the MycoAlert mycoplasma detection kit (Lonza Group Ltd., Basel, Switzerland) at the time of thawing the frozen cell lines and were authenticated by short tandem repeat profiling in the Characterized Cell Line Core at the University of Texas MD Anderson Cancer Center.

### 4.5. Stable Transfectants

To generate ARK1 cells with doxycycline-inducible UCHL1 knockdown, luciferase-labelled cells were transduced with TRIPZ inducible lentiviral non-silencing control (ARK1-luc-dox-NT) (RHS4743, Dharmacon, Lafayette, CO, USA), or anti-UCHL1 shRNA (ARK1-luc-dox-shUCHL1) (RHS4696-200764988, Dharmacon) and selected for with puromycin. Stable cell lines overexpressing UCHL1 were generated by transfection with custom cDNA encoding human UCHL1 in a pcDNA3.1 vector (GenScript, Piscataway, NJ, USA), and selected for with G418.

### 4.6. Antibodies and Reagents

Anti-cyclin B1 from Santa Cruz (sc-245) was used for immunohistochemistry, western blot, immunoprecipitation, immunofluorescence and the proximity ligation assay. Anti-UCHL1 from Sigma-Aldrich (HPA005993) was used for immunohistochemistry, immunofluorescence and proximity ligation assay; anti-UCHL1 from Cell Signaling Technology (11896) was used for western blot; anti-UCHL1 from R&D Systems (MAB6007) was used for immunoprecipitation. Additional antibodies used for western blot were anti-cyclin D1 (sc-753, Santa Cruz Biotechnology Inc., Dallas, TX, USA), anti-cyclin E (sc-481, Santa Cruz Biotechnology Inc.), anti-p53 (48818, Cell Signaling Technology, Danvers, MA, USA), anti-ubiquitin (3936, Cell Signaling Technology), anti-β-actin (4967, Cell Signaling Technology), anti-β-catenin (9562, Cell Signaling Technology), anti-GSK3α/β (5676, Cell Signaling Technology), anti-p21 (sc-397, Santa Cruz Biotechnology Inc.), and anti-p27 (sc-529, Santa Cruz Biotechnology Inc.).

UCHL1 was transiently silenced by transfection with Silencer select siRNAs (s14616 and s14618; Life Technologies, Carlsbad, CA, USA) duplexed with Lipofectamine RNAiMAX (Life Technologies) at a final concentration of 5 nM. Non-targeting Silencer Select siRNA was the negative control (Life Technologies).

### 4.7. Immunohistochemical Staining

Immunohistochemical staining was performed on paraffin embedded tissue. Slides were deparaffinized and rehydrated before antigen retrieval in citrate buffer (S2307, Poly Scientific, Bay Shore, NY, USA), in a decloaking chamber (Biocare Medical, Pacheco, CA, USA) at 125 °C for 4 min and 90 °C for 1 min. Slides were then stained with primary and secondary antibody using the Lab Vision Autostainer 360 (Thermo Fisher Scientific, Waltham, MA, USA). Using a light microscope, digital photomicrographs of representative areas were taken for each slide at 10× magnification; the staining intensity for each slide was then quantified at least 3 times using the Image-Pro Plus software (version 17, MediaCybernetics) by drawing around the stained tumor tissue and obtaining an intensity score from 0 (pure black) to 255 (pure white) with background correction. An average value was calculated for each slide and transformed linearly onto a scale of 0 to 1 for further analyses, such that 0 is pure white and 1 is pure black. For mixed USC/EEC tumors, only the serous component was quantified. Slides were also stained with anti-cyclin B1 antibody, and the percentage of tumor cells positive for cyclin B1 was determined by visual observation under a light microscope.

### 4.8. Quantitative RT-PCR

Total RNA was extracted from adherent cells using the PureLink RNA mini kit (Life Technologies) per the manufacturer’s instructions. Total RNA (1 μg per sample) was subjected to reverse transcription for the synthesis of single-stranded cDNA using the high-capacity cDNA reverse transcription kit (Applied Biosystems, Foster City, CA, USA). Real-time PCR detection was performed using the TaqMan universal master mix (no uracil-N-glycosylase; Applied Biosystems), PPIA-, UCHL1- and cyclin B1-specific TaqMan gene expression assays (Hs04194521_s1, Hs00985157_m1 and Hs01030099_m1, Applied Biosystems) and the CFX96 Touch real-time PCR detection system (Bio-Rad Laboratories, Hercules, CA, USA). PPIA expression levels were used as a reference to quantify UCHL1 and CCNB1 expression levels via the relative standard curve method.

### 4.9. Western Blot Analysis

Adherent cell cultures were washed twice with PBS and lysed with RIPA buffer (150 mM NaCl, 1.0% IGEPAL^®^ CA-630, 0.5% sodium deoxycholate, 0.1% SDS, 50 mM Tris, pH 8.0) (Sigma-Aldrich, St. Louis, MO, USA) supplemented with protease inhibitor cocktail (Sigma-Aldrich). Protein concentration was determined by the BCA assay (Pierce, Appleton, WI, USA). Protein lysates were separated on SDS-polyacrylamide gel and electrophoretically transferred to PVDF membrane (Bio-Rad Laboratories). Membranes were incubated with primary antibodies overnight at 4 °C, then incubated with the HRP-conjugated secondary antibodies (GE Healthcare Life Sciences, Pittsburgh, PA, USA) for 1 h at room temperature. Signals were developed using the HyGlo quick spray chemiluminescent kit (Denville Scientific, Swedesboro, NJ, USA) and visualized on autoradiography film (Denville Scientific). Relative protein levels were quantified with ImageJ (version 1.50i, National Institutes of Health, Bethesda, MD, USA) by normalizing to β-actin.

### 4.10. MTT Assay

Cell proliferation was measured by the MTT assay. Cell cultures were incubated for 2 h at 37 °C in 0.5 mg/mL thiazolyl blue tetrazolium bromide (MTT) in medium, after which the mixture was aspirated and replaced with DMSO. Absorbance was measured at 590 nm using a FLUOstar Omega plate reader (BMG Labtech, Offenburg, Germany). Experiments were performed in triplicate.

### 4.11. In Vivo UCHL1 Knockdown Study

Six-week old, female nude mice were injected intraperitoneally with 6 × 10^6^ ARK1-luc-dox-shNT cells or ARK1-luc-dox-shUCHL1 cells. One day after injection, mice were switched to an irradiated diet containing 625 mg/kg doxycycline (TD.01306, Harlan, Indianapolis, IN, USA). Mice were sacrificed when moribund and tumors were collected for immunohistochemical analysis. The described animal study have been reviewed and approved by the institutional animal care and use committee (IACUC) of the MD Anderson Cancer Center (The ethical code of the animal protocol is 00000675-RN02). 

### 4.12. In Vivo LDN-57444 Treatment

Six-week old, female nude mice were injected intraperitoneally with 6 × 10^6^ ARK1-luc-dox-shNT cells cultured without doxycycline. After 2 weeks, mice were assigned randomly to the two groups and treated thrice weekly by intraperitoneal injection of 0.5 mg/kg LDN-57444 (Tocris Bioscience, Bristol, UK) or control PBS with solvent (0.5% DMSO and 2.5% ethanol). Treatment continued until mice became moribund and were sacrificed. The described animal study have been reviewed and approved by the institutional animal care and use committee (IACUC) of the MD Anderson Cancer Center (The ethical code of the animal protocol is 00000675-RN02).

### 4.13. In Vivo Bioluminescence Imaging

Imaging of tumors was performed using the IVIS Lumina XR imaging system (Caliper Life Sciences, Waltham, MA, USA) and signal quantification was done using Living Image Software (Caliper Life Sciences). Mice were injected intraperitoneally with 120 mg/kg of luciferin, followed by induction of anesthesia with 2–3% isoflurane gas. Bioluminescent images were acquired 10 min after injection.

### 4.14. Immunoprecipitation and Co-Immunoprecipitation

For co-immunoprecipitation studies, cells were harvested in Pierce IP lysis buffer (87787, Thermo Fisher Scientific). 1–2 mg of protein lysate were precleared with 20ul Protein A/G beads (sc-2003, Santa Cruz) for 60 min at 4 °C, then incubated with 4 μg antibody overnight at 4 °C. 40 uL Protein A/G beads were added the next day to pull down bound protein-antibody complexes, and proteins were eluted from the beads with denaturing SDS buffer. For western blot analyses, TrueBlot HRP-conjugated anti-rabbit and anti-mouse antibodies (18-8816-33 and 18-8817-33, Rockland, Limerick, PA, USA) were used to minimize detection of light- and heavy-chain antibody fragments. For detection of cyclin B1 ubiquitination, cells were pretreated with 25 μg/mL MG132 for 4 h before harvesting in RIPA buffer with protease inhibitor and PR-619. As cyclin B1 may be targeted for degradation via K48-ubiquitination, K11-ubiquitination or multiple-monoubiquitination [40,41] total ubiquitin levels were measured by western blot of immunoprecipitated cyclin B1. Ubiquitin staining was quantified with ImageJ (version 1.50i, National Institutes of Health) and normalized to cyclin B1 staining following immunoprecipitation (IP: cyclin B1–WB: cyclin B1).

### 4.15. Immunofluorescence and Proximity Ligation Assay (PLA)

Cells were plated on coated glass slides overnight before methanol fixation. Slides were incubated with primary antibodies in 1% BSA. For immunofluorescence staining, secondary antibodies used were Alexa Fluor 488 anti-rabbit and Alexa Fluor 596 anti-mouse (A-11032, A-11034, Thermo Fisher Scientific). For detection of UCHL1-cyclin B1 protein interaction by PLA, incubation with primary antibody was followed by use of the Duolink In Situ kit (DUO92101, Sigma-Aldrich) per manufacturer’s instructions. Cells were visualized on the Olympus FV1000 laser confocal microscope. To quantify the number of PLA dots in ImageJ (version 1.50i, National Institutes of Health), images were converted to 16-bit grayscale, adjusted for brightness/contrast, and despeckled before analysis with the SpotCounter plugin (version 0.14). The number of dots was divided by number of nuclei to obtain dots/cell.

### 4.16. Cycloheximide Chase Assay

ARK1 and ARK2 cells were treated with 20 µg/mL and 10 µg/mL CHX respectively before cell lysis at the indicated time points for analysis by western blot. Cyclin B1 protein expression levels were determined relative to zero hours of cycloheximide treatment.

### 4.17. TP53 DNA Sequencing

Using DNA extracted from ARK1 and ARK2 cells, exon 5–8 of the TP53 gene were amplified by PCR. The primers used were as follows: exon 5–6, forward 5′-TGTTCACTTGTGCCCTGACT-3′, reverse 5′-GAGGGCCACTGACAACCA-3′; exon 7, forward 5′-AGGTCTCCCCAAGGCGCACTG-3′, reverse 5′-TGTGCAGGGTGGCAAGTGGC-3′; exon 8, forward 5′-TGGGAGTAGATGGAGCCTGG-3′, reverse 5′-AGGAAAGAGGCAAGGAAAGG-3′. Amplified DNA fragments were analyzed by Sanger sequencing.

### 4.18. Apoptosis Assay

Apoptosis was measured in ARK1 cells by using the Dead Cell Apoptosis Kit (V13241, Invitrogen) per the manufacturer’s instructions. Cells were stained with Alexa Fluor 488 annexin V and propidium iodide, then read a Gallios flow cytometer (Beckman Coulter, Brea, CA, USA). Cells with both propidium iodide and annexin V were considered apoptotic.

### 4.19. Migration Assay

Three days after siRNA transfection, ARK1 and ARK2 cells were serum starved for 24 h; equal numbers were then plated into migration chambers with 8 µm pore size, (3422, Corning) in serum-free medium. Medium supplemented with 20% fetal-bovine serum was placed below the chamber. Cells were allowed to migrate for 24 h before fixation with ice-cold methanol and staining with crystal violet solution.

### 4.20. Cell Cycle Analysis by Flow Cytometry

Adherent cells were trypsinized, washed once with PBS, and fixed overnight at 4 °C with 70% ethanol. Cells were then incubated with 0.1 mg/mL RNase A solution and 50 μg/mL propidium iodide at 37 °C for 30 min. Stained cells were subsequently analyzed using a Gallios flow cytometer (Beckman Coulter) to determine the percentage of cells in the G1, S and G2/M phases.

### 4.21. Statistical Analysis

Survival analysis was performed with SPSS software version 19. Statistical analysis was performed with SPSS and GraphPad PRISM version 6. Two-tailed comparisons were performed using *T*-Test, Mann-Whitney *U*-test or Kruskal-Wallis *H*-test as appropriate. Post-hoc testing for nonparametric pairwise multiple comparisons was performed with the Dunn’s test. Adjusted *p*-values for multiple comparisons was calculated by *p* × *n* × (*n* − 1)/2, where *n* is the number of independent groups. Correlation between variables was tested using the Spearman’s test.

## 5. Conclusions

Taken together, our findings suggest that UCHL1 contributes to USC tumorigenesis by stabilizing cyclin B, promoting cell cycle progression and tumor growth, and contributing to poor patient survival rates in USC patients. Therefore, UCHL1 is a novel prognostic marker for USC and a viable therapeutic target. Although several compounds, including LDN-57444 [20,42,43], have been identified as UCHL1 inhibitors, their role is currently restricted as research tools. The development of additional UCHL1 inhibitors with increased specificity and vehicles to deliver the drug specifically to the tumor may increase the efficacy and lower the toxicity of the drug for clinical use.

## Supplementary Materials

The following are available online at https://www.mdpi.com/2072-6694/12/1/118/s1, Figure S1: UCHL1 expression is higher in omental metastasis compared to primary tumor and is associated with poorer disease-free and overall survival in late-stage USC patients, Figure S2: UCHL1 affects tumor cell proliferation in vitro and in vivo, Figure S3: The effect of UCHL1 silencing on the protein levels of genes involved in cell cycle and proliferation, Figure S4: Correlation between UCHL1 RNA expression and major cyclins’ protein expressions, Figure S5: UCHL1 and cyclin B1 colocalize and interact in vivo, Figure S6: UCHL1 affects the progression of cells through cell cycle and is associated with aneuploidy score in endometrial tumors, Table S1: 19 candidate genes with higher expression in USC than in normal tissue and low-grade EEC, and associated with poorer overall survival in USC patients, Table S2: Cox regression analysis of overall survival of USC patients in the TCGA data set, Table S3: Demographic characteristics of patients in the immunohistochemical analysis of UCHL1, Table S4: Cox regression analysis of late-stage USC patients with no evidence of disease after treatment.
