# Supplementary material for: Ubiquitin Carboxyl-Terminal Hydrolase L1 (UCHL1) Promotes Uterine Serous Cancer Cell Proliferation and Cell Cycle Progression
Source: Cancers (Basel). 2020 Jan 2;12(1):118. doi: 10.3390/cancers12010118 (PMC7016780; doi:10.3390/cancers12010118)
Supplement: Supplementary file 1 [file cancers-12-00118-s001.zip › cancers-679705-suppl-final.docx.docx]

Supplemental Materials: Ubiquitin Carboxyl-Terminal Hydrolase L1 (UCHL1) Promotes Uterine Serous Cancer Cell Proliferation and Cell Cycle Progression

Suet-Ying Kwan, Chi-Lam Au-Yeung, Tsz-Lun Yeung, Angela Rynne-Vidal, Kwong-Kwok Wong, John I. Risinger, Hui-Kuan Lin, Rosemarie E. Schmandt, Melinda S. Yates, Samuel C. Mok and Karen H. Lu


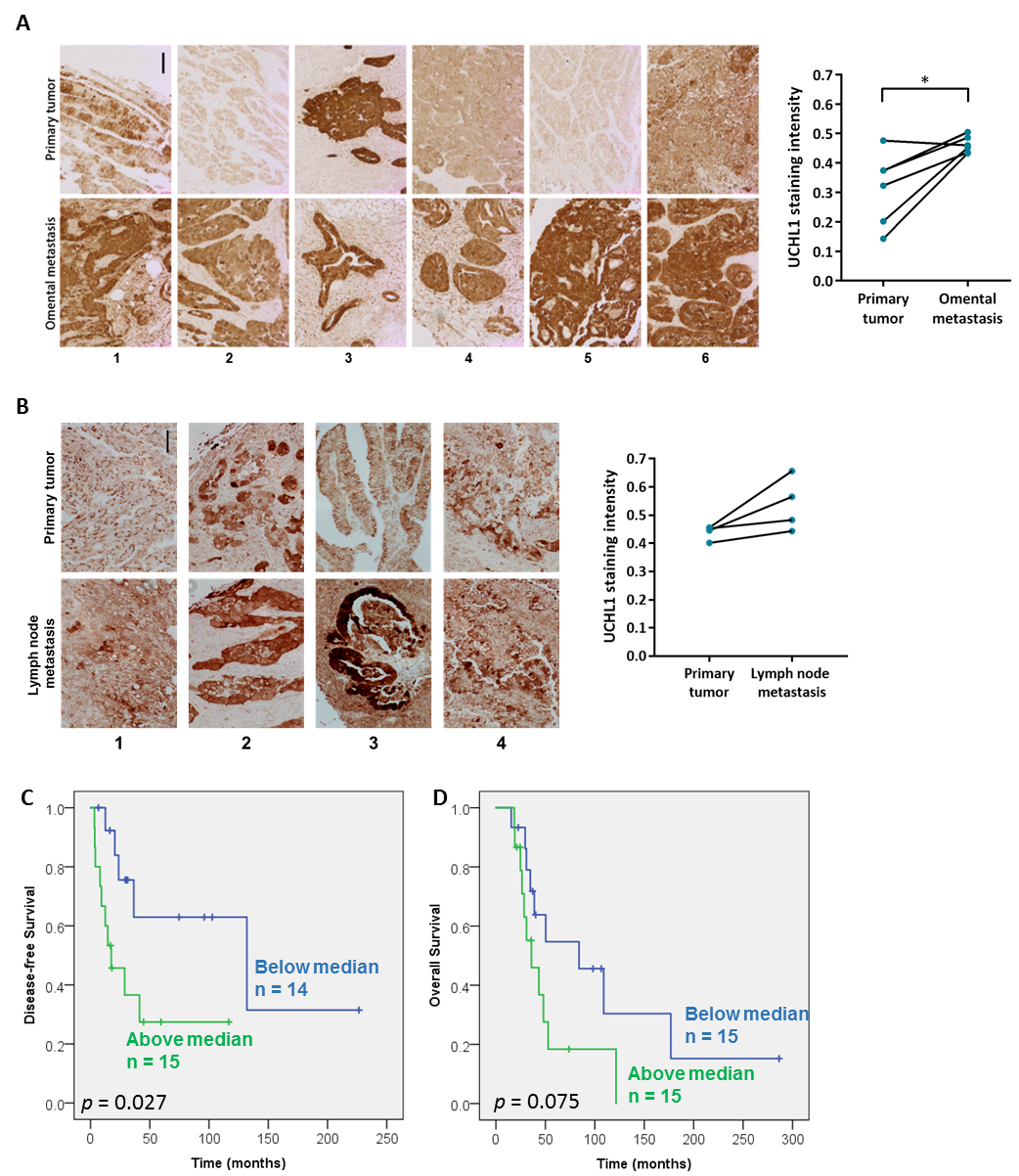


**Figure S1.** UCHL1 expression is higher in metastasis compared to primary tumor and is associated with poorer disease-free and overall survival in late-stage USC patients.
Immunohistochemical staining of UCHL1 in paired primary tumor and (**A**) omental metastases samples from patients 1–6 or (**B**) lymph node metastases samples from patients 1–4. Scale bar, 100 µm. UCHL1 staining intensity of paired samples from the same patient is presented on the right. Statistical significance was determined by the paired *T*-test. * *p* < 0.05. Kaplan-Meier analysis of (**C**) disease-free and (**D**) overall survival of USC patients in the validation cohort, grouped above and below the median UCHL1 staining intensity. Analysis is of late-stage USC patients with no evidence of disease at time of treatment end. Cases with missing survival data have been excluded. Statistical significance was determined by the log-rank test.


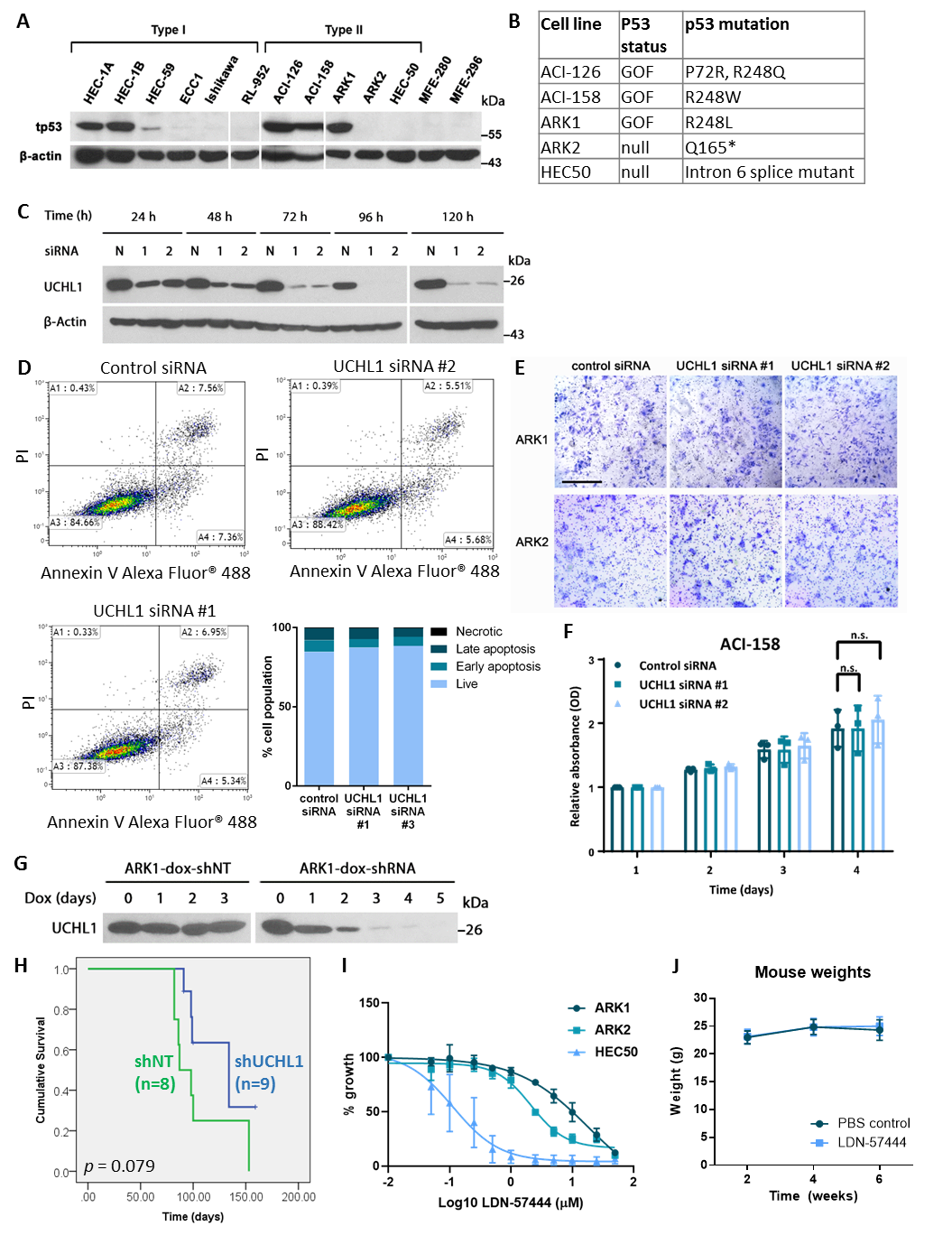


**Figure 2.** UCHL1 affects tumor cell proliferation in vitro and in vivo. (**A**,**B**) p53 protein expression in the endometrial cancer cell line panel, and p53 mutation status in type II cell lines. (**C**) Silencing of UCHL1 in ARK1 cells following transfection with UCHL1 siRNA #1 and #2. (**D**) Apoptosis was measured in ARK1 cells four days after transfection with control or UCHL1-silencing siRNA by labelling with annexin V Alexa Fluor 488 and propidium iodide. (**E**) Four days after siRNA transfection, equal numbers of ARK1 and ARK2 cells were seeded onto 8 µm transwell inserts. 24 h later, migrated cells were visualized by crystal violet staining. Scale bar, 500 µm. (**F**) Effect of UCHL1 knockdown on cell proliferation in ACI-158 cells in vitro, as measured by the MTT assay. Statistical significance was determined by the *T*-Test (two-tailed, equal variance). n.s. = not significant. Error bars represent mean ± SD (3 biological replicates). (**G**) UCHL1 protein levels in ARK1 cells stably transduced with doxycycline-inducible control shRNA or UCHL1 shRNA, and cultured in medium with 2µg/mL doxycycline. (**H**) Kaplan-Meier survival curves of nude mice injected with ARK1 cells transduced with doxycycline-inducible control shRNA or anti-UCHL1 shRNA. Statistical significance was determined by the log-rank test. (**I**) Survival curves of ARK1, ARK2 and HEC-50 cells after 72-h treatment of LDN-57444. Error bars represent mean ± SD. (**J**) Mouse weights of nude mice following treatment with PBS control (0.5% DMSO, 2.5% ethanol) or 0.5 mg/kg LDN-57444. Error bars represent mean ± SD.


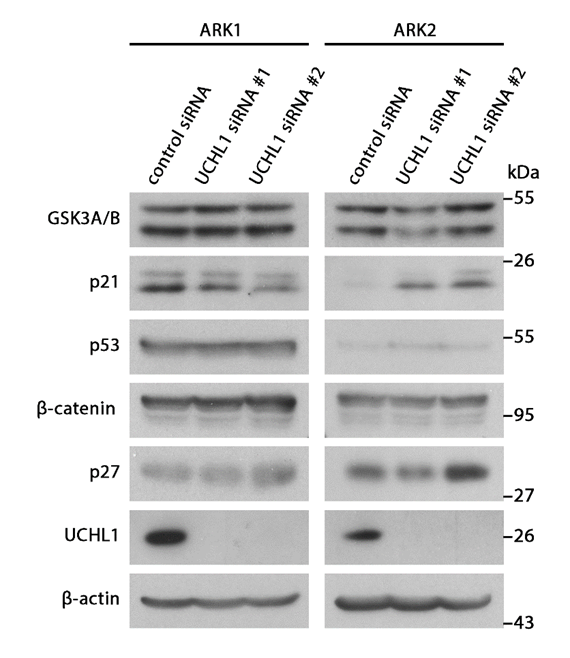


**Figure S3.** The effect of UCHL1 silencing on the protein levels of genes involved in cell cycle and proliferation. Western blot was performed four days after transfection with control or anti-UCHL1 siRNA in ARK1 and ARK2 cells.


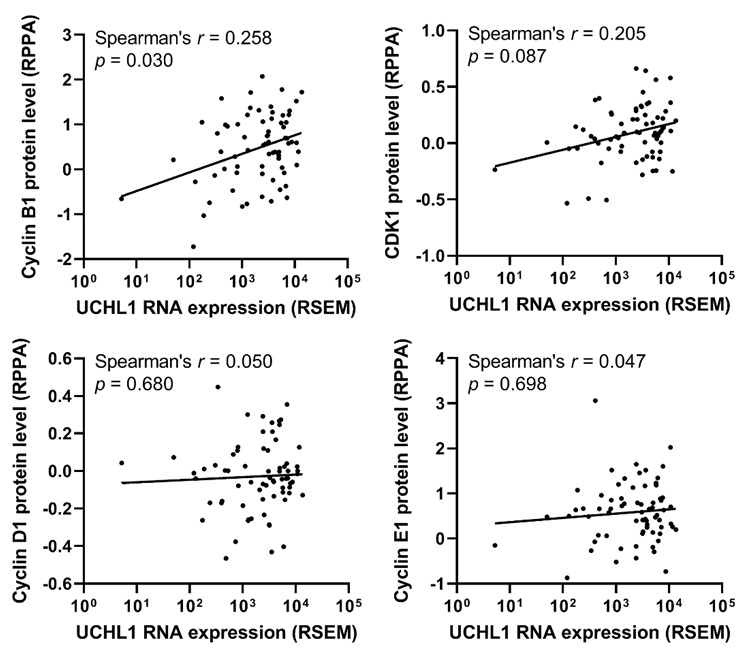


**Figure S4.** Correlation between UCHL1 RNA expression and major cyclins’ protein expressions. Scatter plots of UCHL1 RNA expression versus protein expression of cyclin B1, CDK1, cyclin D1 and cyclin E1, in USC tumors of the TCGA data set (*n* = 71).


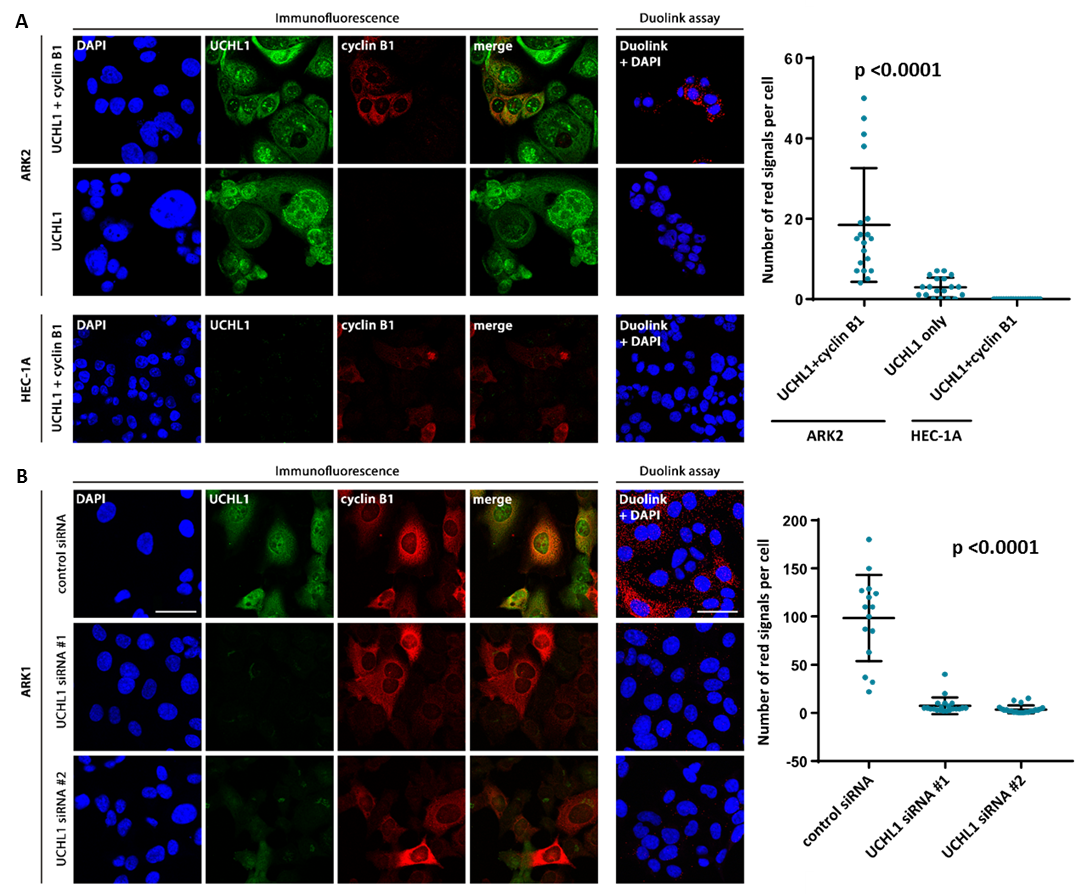


**Figure S5.** UCHL1 and cyclin B1 colocalize and interact in vitro. Immunofluorescence staining and the Duolink proximity ligation assay was performed on (**A**) ARK2 cells and the UCHL1-negative HEC-1A cells. As a control, ARK2 cells were also stained with anti-UCHL1 antibody only. (**B**) ARK1 cells 4 days after transfection with control siRNA or anti-UCHL1 siRNA. Following the Duolink assay, red fluorescent dots indicate presence of protein-protein interaction. Scale bar, 50 µm. Results in the dot plot show the number of red signals per cell in three independent experiments. Statistical significance was determined by the Kruskal-Wallis test. Error bars represent mean ± SD (*p* < 0.0001).


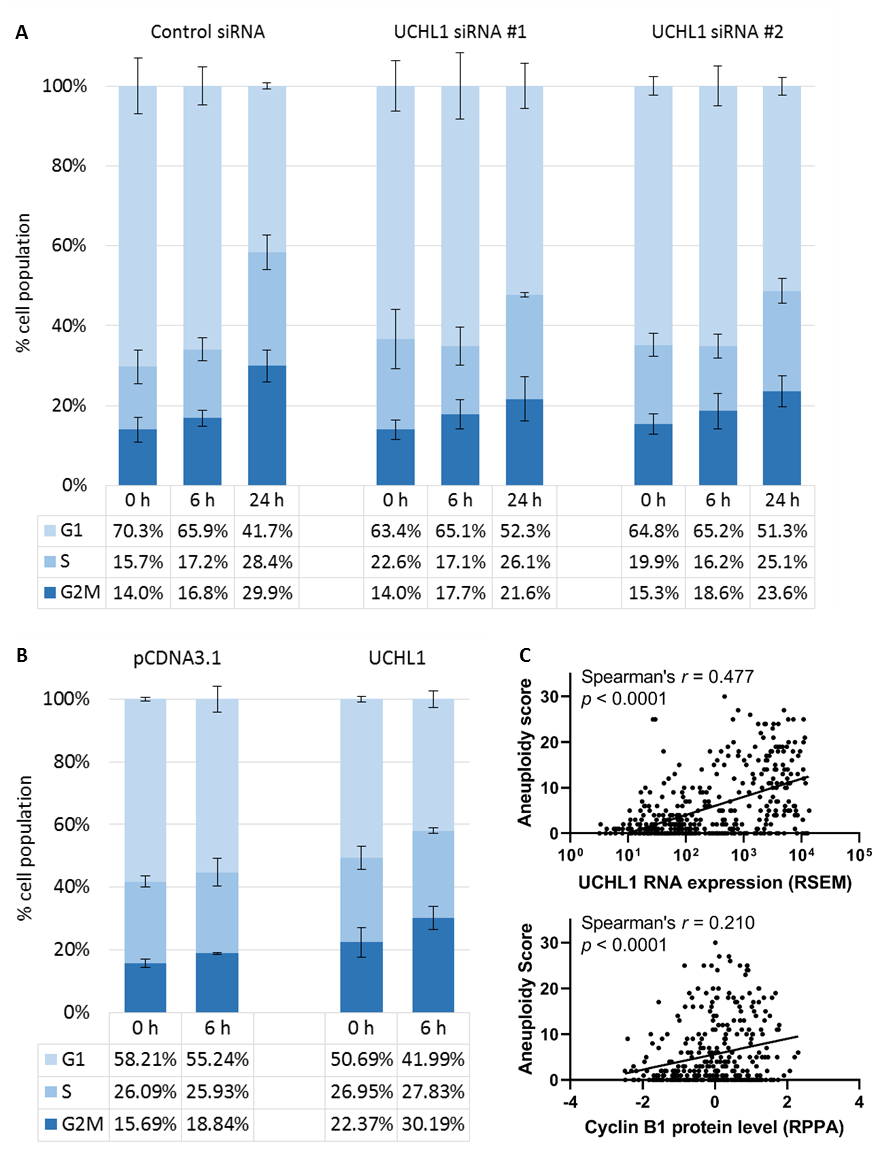


**Figure S6.** UCHL1 affects the progression of cells through the cell cycle and is associated with aneuploidy score in endometrial tumors. ARK1 cells transfected with anti-UCHL1 siRNA (*p* < 0.05; *p* = 0.066) (**A**) and HEC-50 cells overexpressing UCHL1 (*p* < 0.05) (**B**) were serum starved for 24-h before release into medium with 10% FBS; cells were collected for flow cytometry analysis at the indicated time points. Error bars represent mean ± SD (3 biological replicates). (**C**) UCHL1 RNA expression and cyclin B1 protein expression are significantly correlated with aneuploidy score in endometrial tumors of the TCGA PanCancer Atlas data set (*n* = 425).

**Table S1.** 19 candidate genes with higher expression in USC than in normal tissue and low-grade EEC, and associated with poorer overall survival in USC patients.

| **Gene** | **Entrez ID** | **Median RSEM Expression** | | | **SAM: USC vs. Normal** | | | **SAM: USC vs. EEC (G1/2)** | | | **Log-Rank Test** | |
| --- | --- | --- | --- | --- | --- | --- | --- | --- | --- | --- | --- | --- |
|  |  | **Normal** | **EEC (G1/2)** | **USC** | ***q*** | ***d*** | **Fold Change** | ***q*** | ***d*** | **Fold Change** | ***p*** | **HR** |
| UCHL1 | 7345 | 166 | 60 | 2780 | 0.000 | 776.3 | 15.3 | 0.000 | 7130.9 | 40.9 | 0.023 | 2.64 |
| WASF1 | 8936 | 346 | 354 | 928 | 0.000 | 615.4 | 2.5 | 0.000 | 5003.2 | 2.4 | 0.045 | 2.43 |
| CCDC99 | 54908 | 154 | 218 | 506 | 0.000 | 860.7 | 2.9 | 0.000 | 5553.2 | 2.1 | 0.014 | 2.86 |
| PLXNA4 | 91584 | 119 | 76 | 283 | 0.050 | 187.8 | 2.2 | 0.000 | 2431.8 | 3.5 | 0.020 | 3.12 |
| COL9A1 | 1297 | 3 | 15 | 239 | 0.000 | 698.0 | 75.1 | 0.000 | 4530.3 | 14.5 | 0.039 | 2.50 |
| FAM167A | 83648 | 23 | 54 | 150 | 0.000 | 529.0 | 6.1 | 0.000 | 3225.0 | 2.6 | 0.004 | 3.51 |
| MMP10 | 4319 | 1 | 42 | 115 | 0.000 | 609.7 | 92.2 | 0.006 | 1582.8 | 2.8 | 0.047 | 2.44 |
| MMP1 | 4312 | 1 | 39 | 86 | 0.000 | 871.6 | 73.5 | 0.000 | 2355.5 | 2.1 | 0.001 | 4.11 |
| C11orf41 | 25758 | 6 | 15 | 54 | 0.000 | 632.4 | 7.4 | 0.000 | 3038.9 | 3.3 | 0.021 | 2.70 |
| PPAPDC1A | 196051 | 5 | 10 | 37 | 0.000 | 785.3 | 8.2 | 0.000 | 3584.5 | 3.4 | 0.047 | 2.36 |
| NKAIN1 | 79570 | 4 | 8 | 33 | 0.000 | 731.6 | 8.3 | 0.000 | 4266.3 | 3.9 | 0.008 | 3.28 |
| GAL | 51083 | 0 | 6 | 27 | 0.000 | 738.5 | 3.8 × 10^9^ | 0.000 | 4131.0 | 4.4 | 0.030 | 2.75 |
| SLCO4C1 | 353189 | 3 | 5 | 17 | 0.000 | 589.2 | 4.8 | 0.000 | 3818.0 | 3.3 | 0.038 | 2.53 |
| FOXE1 | 2304 | 0 | 0 | 4 | 0.000 | 683.9 | 5.3 × 10^8^ | 0.000 | 4129.4 | 4.1 × 10^8^ | 0.030 | 2.75 |
| DDC | 1644 | 1 | 1 | 4 | 0.000 | 448.1 | 3.7 | 0.000 | 2563.7 | 3.2 | 0.018 | 2.99 |
| NR5A1 | 2516 | 0 | 0 | 1 | 0.006 | 310.3 | 1.2 × 10^8^ | 0.000 | 2977.9 | 9.6 × 10^7^ | 0.009 | 3.26 |
| TBX22 | 50945 | 0 | 0 | 1 | 0.001 | 387.9 | 1.3 × 10^8^ | 0.041 | 1072.0 | 9.8 × 10^7^ | 0.032 | 2.53 |
| LOC400940 | 400940 | 0 | 0 | 1 | 0.023 | 239.2 | 8.0 × 10^7^ | 0.000 | 2758.1 | 6.1 × 10^7^ | 0.008 | 3.27 |
| DAD1L | 56286 | 0 | 0 | 0 | 0.009 | 292.3 | 5.9 × 10^7^ | 0.003 | 1720.2 | 4.5 × 10^7^ | 0.040 | 2.41 |

Genes are ordered by median RSEM expression in USC patients. SAM analysis was performed with level 3 raw counts from the RNA sequencing dataset. The SAM score (*d*) is the relative difference in gene expression. Hazard ratio is the risk of patients with gene expression above the median divided by the risk of patients with gene expression below the median. *q* = q-value; *d* = SAM score; *p* = *p*-value; HR = hazard ratio.

**Table S2.** Cox regression analysis of overall survival of USC patients in the TCGA data set.

| **Variable** | | **Pts (*n*)** | **Events (*n*)** | **HR** | **95% CI** | ***p*** |
| --- | --- | --- | --- | --- | --- | --- |
| Age | |  |  |  |  |  |
|  |  | 69 | 15 | 1.07 | 0.99–1.17 | 0.107 |
| Stage * | |  |  |  |  |  |
|  | I | 29 | 4 | 1.00 | Reference | |
|  | II | 5 | 1 | 0.59 | 0.05–5.73 | 0.587 |
|  | III | 30 | 8 | 1.57 | 0.58–9.34 | 0.230 |
|  | IV | 5 | 2 | 3.07 | 1.61–211.97 | 0.019 ^a^ |
| Residual tumor * | |  |  |  |  |  |
|  | R0 | 55 | 10 | 1.00 | Reference | |
|  | R1-R2 | 14 | 5 | 0.88 | 0.23–3.44 | 0.855 |
| UCHL1 expression * | |  |  |  |  |  |
|  | Below median | 36 | 6 | 1.00 | Reference | |
|  | Above median | 33 | 9 | 3.24 | 1.01–10.44 | 0.049 ^a^ |

Cases with missing information were excluded from analysis. R0 = no residual tumor; R1 = microscopic residual tumor; R2 = macroscopic residual tumor; Pts = patients; HR = hazard ratio; CI = confidence intervals; *p* = *p*-value; * = categorical variable; a = statistically significant.

**Table S3.** Demographic characteristics of patients in the immunohistochemical analysis of UCHL1.

| **Parameter** | | **Endometrioid (*n* = 34)** | **Serous (*n* = 80)** | **Total (*n* = 114)** |
| --- | --- | --- | --- | --- |
|  |  | *n* (%) | *n* (%) | *n* (%) |
| **Age, Years** | |  |  |  |
|  | Mean | 64.2 | 68.4 | 67.1 |
|  | Standard deviation | 8.5 | 7.9 | 8.3 |
|  | Median | 63.0 | 69.0 | 67.0 |
|  | Minimum | 53.0 | 43.0 | 43.0 |
|  | Maximum | 83.0 | 85.0 | 85.0 |
| **Ethnicity** | |  |  |  |
|  | Caucasian | 25 (74%) | 50 (63%) | 75 (66%) |
|  | African American | 2 (6%) | 22 (28%) | 24 (21%) |
|  | Hispanic | 7 (21%) | 5 (6%) | 12 (11%) |
|  | Asian | 0 | 3 (4%) | 3 (3%) |
| **Histology Purity** | |  |  |  |
|  | Pure | 34 (100%) | 53 (66%) | 87 (76%) |
|  | Mixed | 0 (0%) | 27 (34%) | 27 (24%) |
| **Grade** | |  |  |  |
|  | 1 | 10 (29%) | 0 | 10 (9%) |
|  | 2 | 15 (44%) | 0 | 15 (13%) |
|  | 3 | 9 (27%) | 80 (100%) | 89 (78%) |
| **Stage** | |  |  |  |
|  | I | 19 (56%) | 31 (39%) | 50 (44%) |
|  | II | 4 (12%) | 3 (4%) | 7 (6%) |
|  | III | 6 (18%) | 28 (35%) | 34 (30%) |
|  | IV | 5 (15%) | 18 (23%) | 23 (20%) |
| **Lymph Node Dissection** | | 0 (0%) | 18 (23%) | 18 (16%) |
| **BMI (kg/m^2^)** | |  |  |  |
|  | Eutrophic (18.50–24.99) | 0 (0%) | 15 (19%) | 15 (13%) |
|  | Overweight (25–29.99) | 6 (18%) | 12 (15%) | 16 (14%) |
|  | Obese (≥30) | 27 (79%) | 40 (50%) | 67 (59%) |
|  | Unknown | 1 (3%) | 13 (16%) | 16 (14%) |
| **UCHL1 Staining Intensity** | |  |  |  |
|  | Mean | 0.26 | 0.35 | 0.32 |
|  | Standard deviation | 0.03 | 0.08 | 0.08 |
|  | Median | 0.26 | 0.35 | 0.29 |
|  | Minimum | 0.20 | 0.22 | 0.20 |
|  | Maximum | 0.39 | 0.56 | 0.56 |

*n* = number of patients.

**Table S4.** Cox regression analysis of late-stage USC patients with no evidence of disease after treatment.

|  |  | **Disease-Free Survival** | | | | | **Overall Survival** | | | | |
| --- | --- | --- | --- | --- | --- | --- | --- | --- | --- | --- | --- |
| **Variable** | | **Pts (*n*)** | **Events (*n*)** | **HR** | **95% CI** | ***p*** | **Pts (*n*)** | **Events (*n*)** | **HR** | **95% CI** | ***p*** |
| Age | |  |  |  |  |  |  |  |  |  |  |
|  |  | 29 | 15 | 1.01 | 0.94–1.08 | 0.853 | 30 | 20 | 1.03 | 0.95–1.10 | 0.512 |
| Histology* | |  |  |  |  |  |  |  |  |  |  |
|  | Pure USC | 22 | 12 | 1.00 | Reference | | 23 | 16 | 1.00 | Reference | |
|  | Mixed: EEC/USC | 7 | 3 | 0.48 | 0.11–2.03 | 0.316 | 7 | 4 | 0.32 | 0.08–1.23 | 0.097 |
| Stage* | |  |  |  |  |  |  |  |  |  |  |
|  | III | 19 | 9 | 1.00 | Reference | | 19 | 12 | 1.00 | Reference | |
|  | IV | 10 | 6 | 1.96 | 0.65–5.93 | 0.231 | 11 | 8 | 2.25 | 0.81–6.24 | 0.119 |
| UCHL1 expression* | |  |  |  |  |  |  |  |  |  |  |
|  | Below median | 14 | 5 | 1.00 | Reference | | 15 | 9 | 1.00 | Reference | |
|  | Above median | 15 | 10 | 4.16 | 1.20–14.37 | 0.024 ^a^ | 15 | 11 | 3.86 | 1.27–11.74 | 0.017 ^a^ |

Cases with missing survival information were excluded from analysis. Pts = patients, HR = hazard ratio; CI = confidence intervals; *p* = *p*-value; * = categorical variable; a = statistically significant.

| 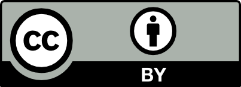 | © 2019 by the authors. Licensee MDPI, Basel, Switzerland. This article is an open access article distributed under the terms and conditions of the Creative Commons Attribution (CC BY) license (http://creativecommons.org/licenses/by/4.0/). |
| --- | --- |
