# Supplementary material for: Ubiquitin Carboxyl-Terminal Hydrolase L1 (UCHL1) Promotes Uterine Serous Cancer Cell Proliferation and Cell Cycle Progression
Source: Cancers (Basel). 2020 Jan 2;12(1):118. doi: 10.3390/cancers12010118 (PMC7016780; doi:10.3390/cancers12010118)
Supplement: Supplementary file 1 [file cancers-12-00118-s001.zip › Uncropped-blots_2019-12-12.pptx]

## Slide 1
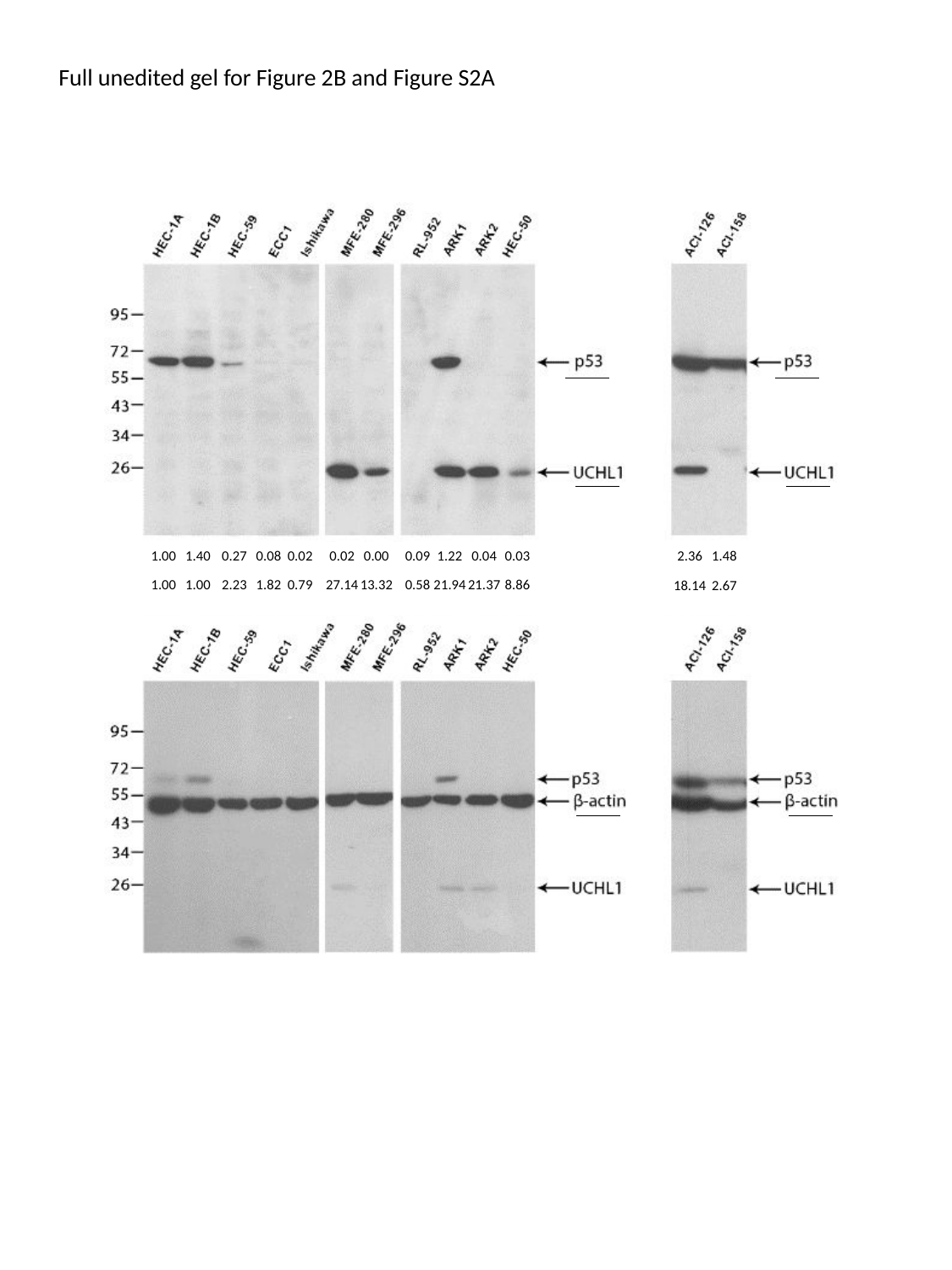

Full unedited gel for Figure 2B and Figure S2A
1.00
1.40
0.27
0.08
0.02
0.02
0.00
0.09
1.22
0.04
0.03
2.36
1.48
1.00
1.00
2.23
1.82
0.79
27.14
13.32
0.58
21.94
21.37
8.86
18.14
2.67

## Slide 2
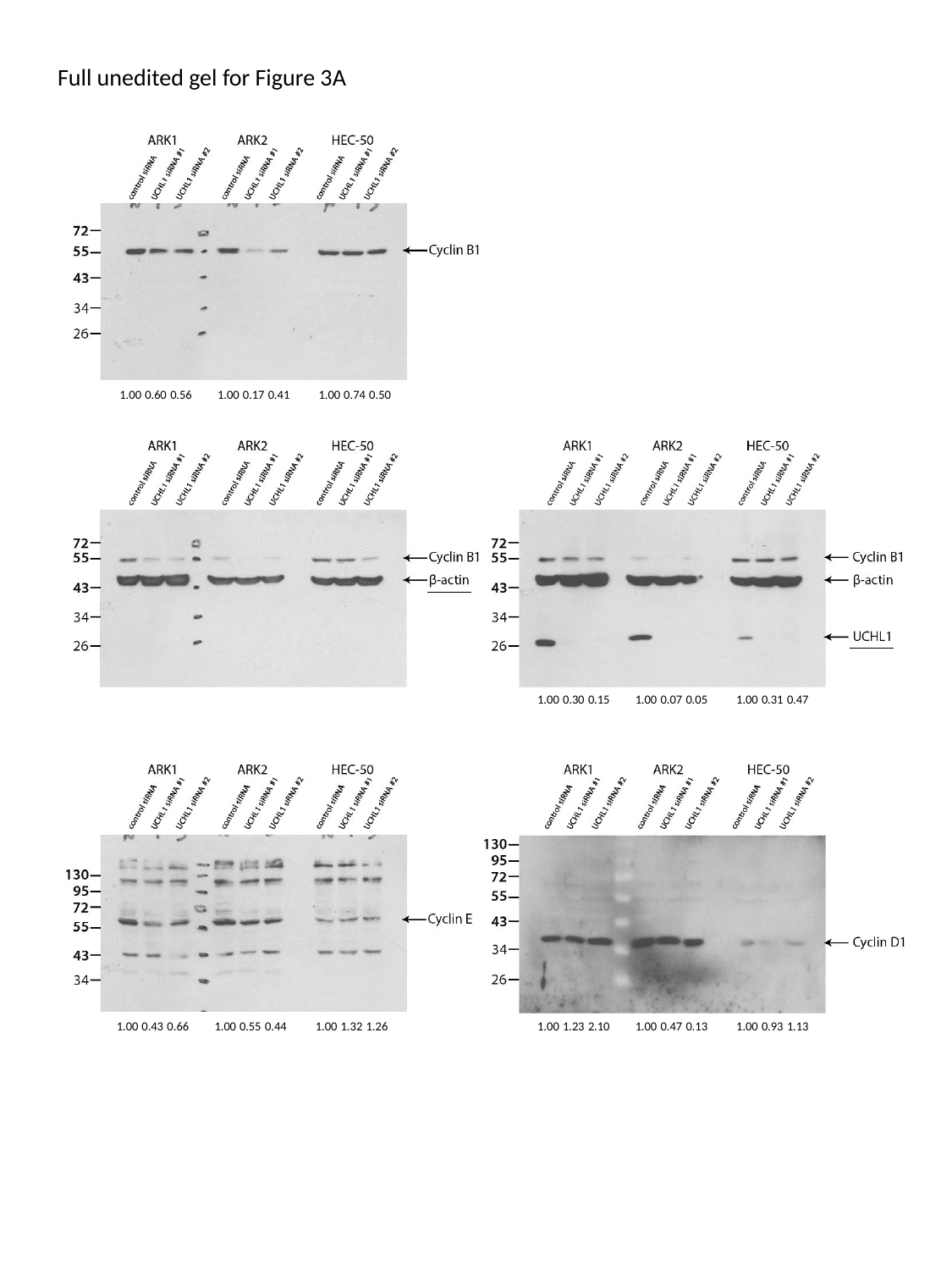

Full unedited gel for Figure 3A
1.00
0.60
0.56
1.00
0.17
0.41
1.00
0.74
0.50
1.00
0.30
0.15
1.00
0.07
0.05
1.00
0.31
0.47
1.00
1.23
2.10
1.00
0.47
0.13
1.00
0.93
1.13
1.00
0.43
0.66
1.00
0.55
0.44
1.00
1.32
1.26

## Slide 3
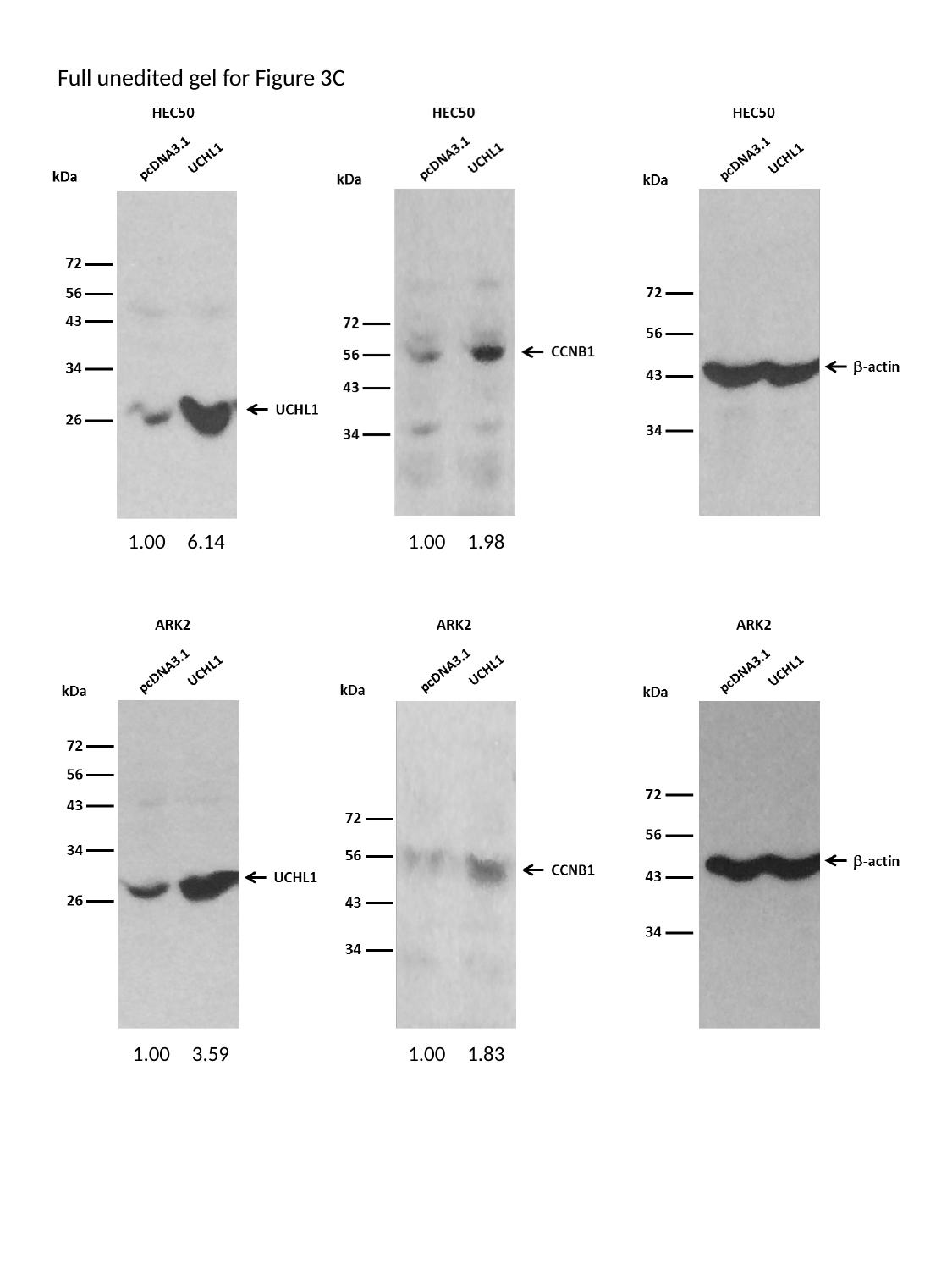

Full unedited gel for Figure 3C
1.00
6.14
1.00
1.98
1.00
3.59
1.00
1.83

## Slide 4
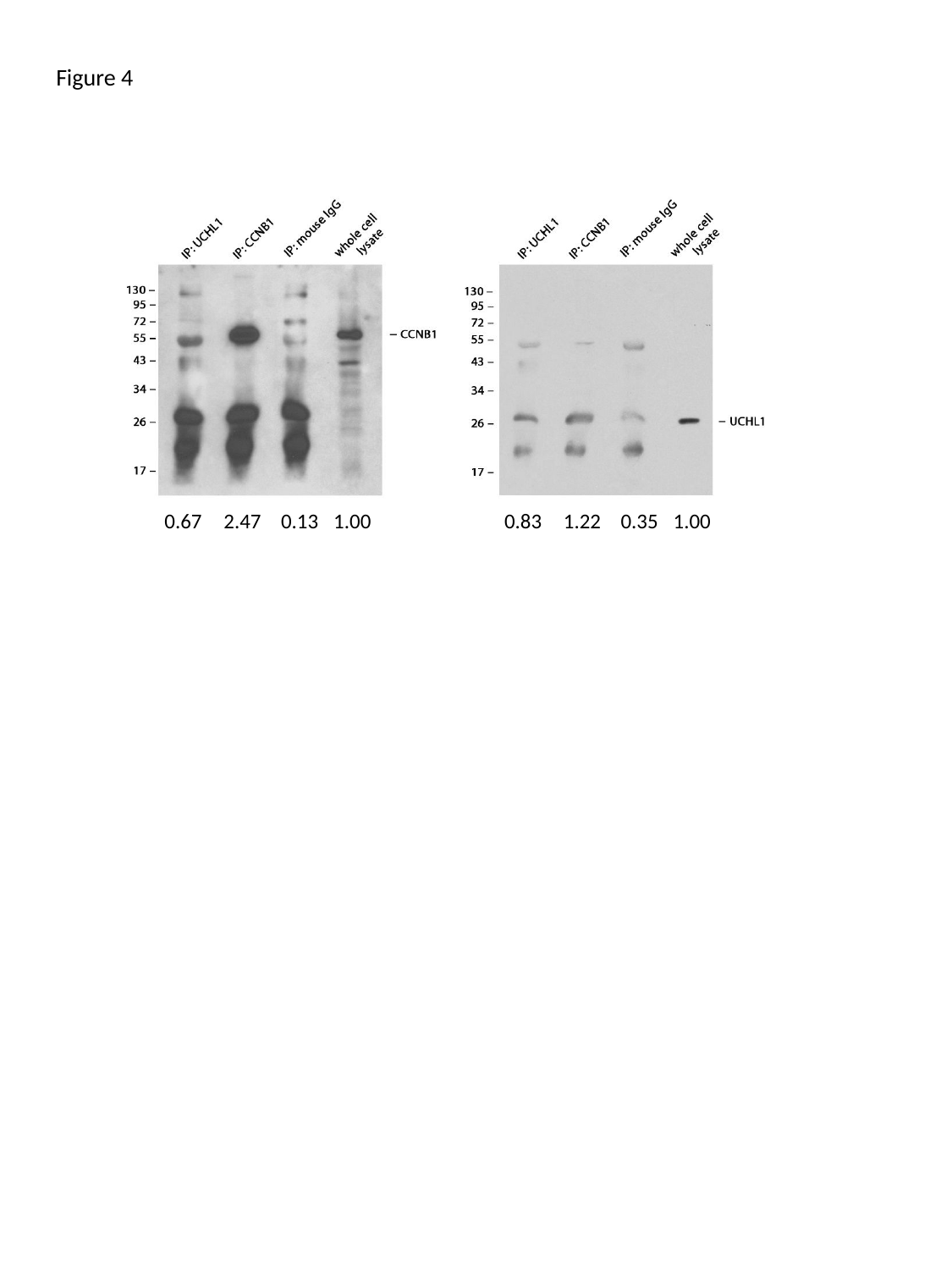

Figure 4
0.67
2.47
0.13
1.00
0.83
1.22
0.35
1.00

## Slide 5
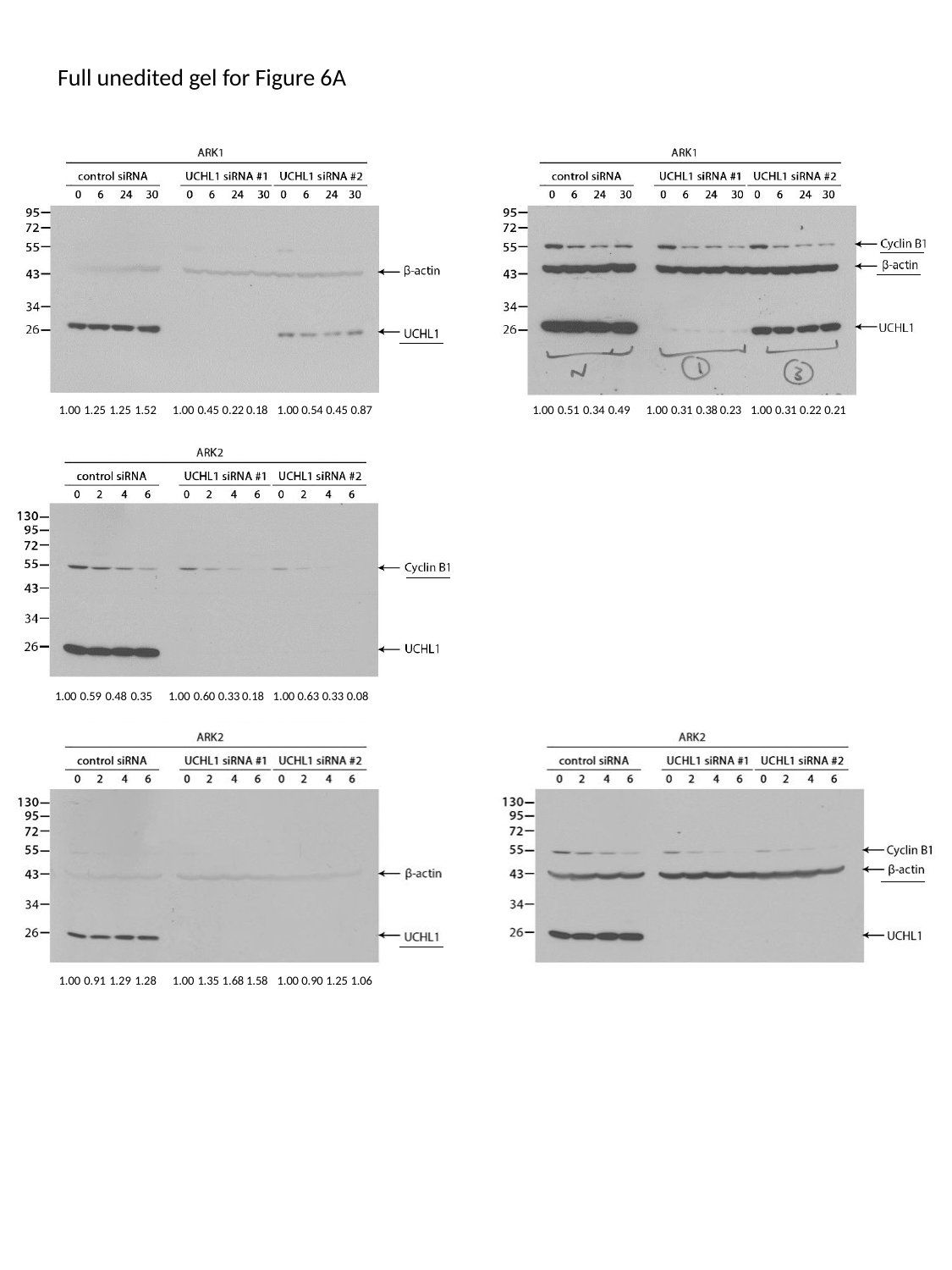

Full unedited gel for Figure 6A
1.00
1.25
1.25
1.52
1.00
0.45
0.22
0.18
1.00
0.54
0.45
0.87
1.00
0.51
0.34
0.49
1.00
0.31
0.38
0.23
1.00
0.31
0.22
0.21
1.00
0.59
0.48
0.35
1.00
0.60
0.33
0.18
1.00
0.63
0.33
0.08
1.00
0.91
1.29
1.28
1.00
1.35
1.68
1.58
1.00
0.90
1.25
1.06

## Slide 6
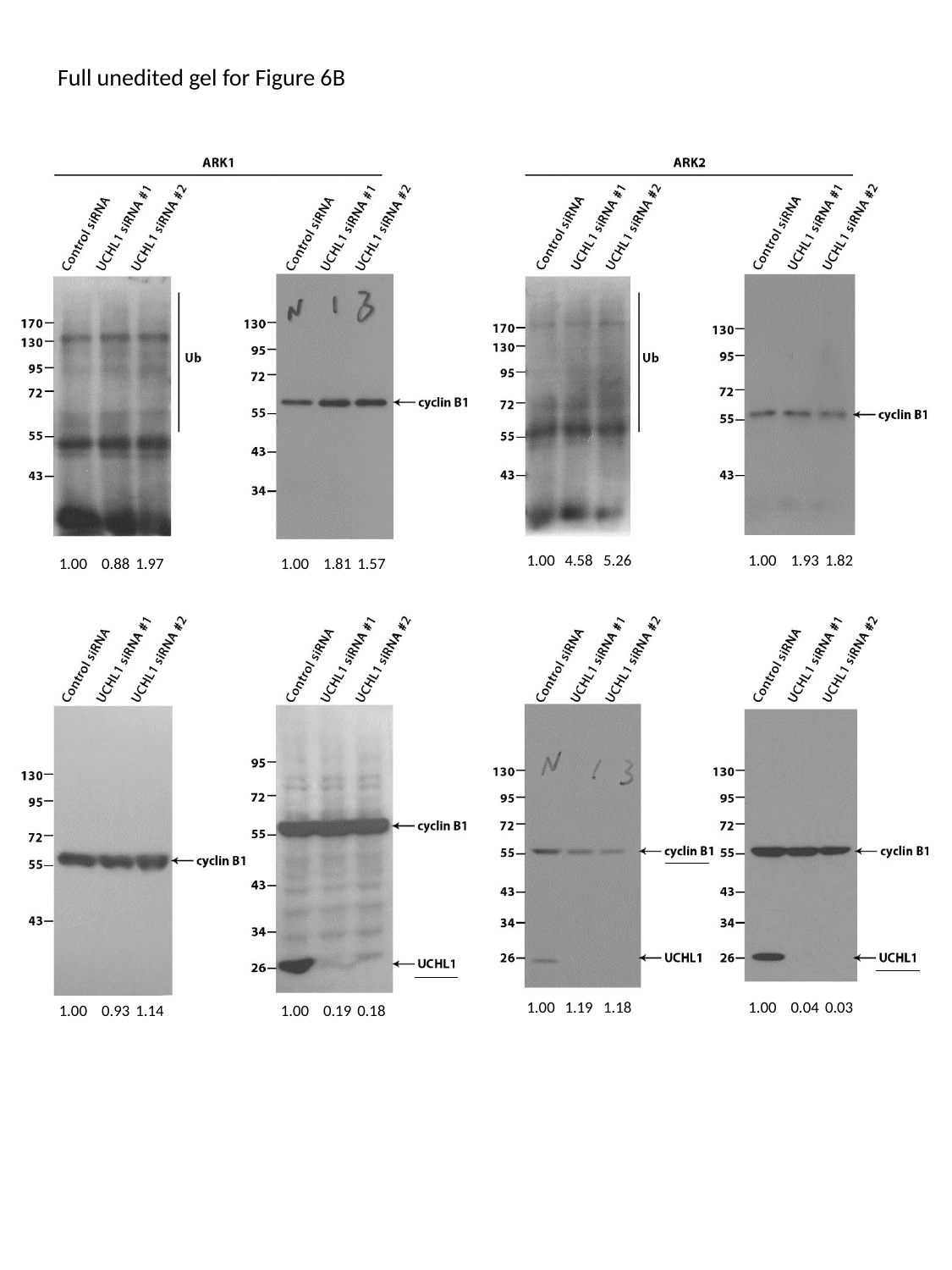

Full unedited gel for Figure 6B
1.00
4.58
5.26
1.00
1.93
1.82
1.00
0.88
1.97
1.00
1.81
1.57
1.00
1.19
1.18
1.00
0.04
0.03
1.00
0.93
1.14
1.00
0.19
0.18

## Slide 7
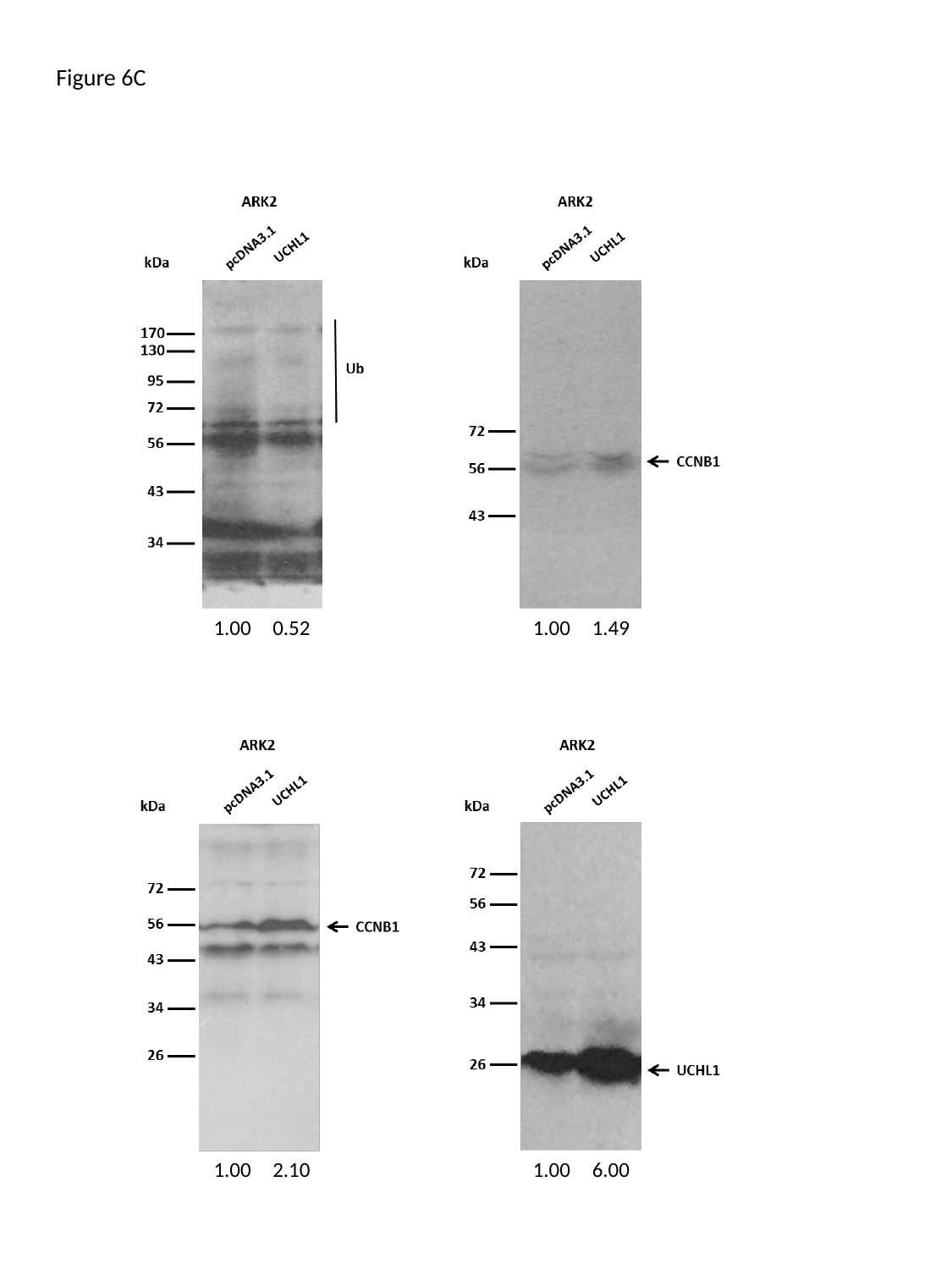

Figure 6C
1.00
0.52
1.00
1.49
1.00
2.10
1.00
6.00

## Slide 8
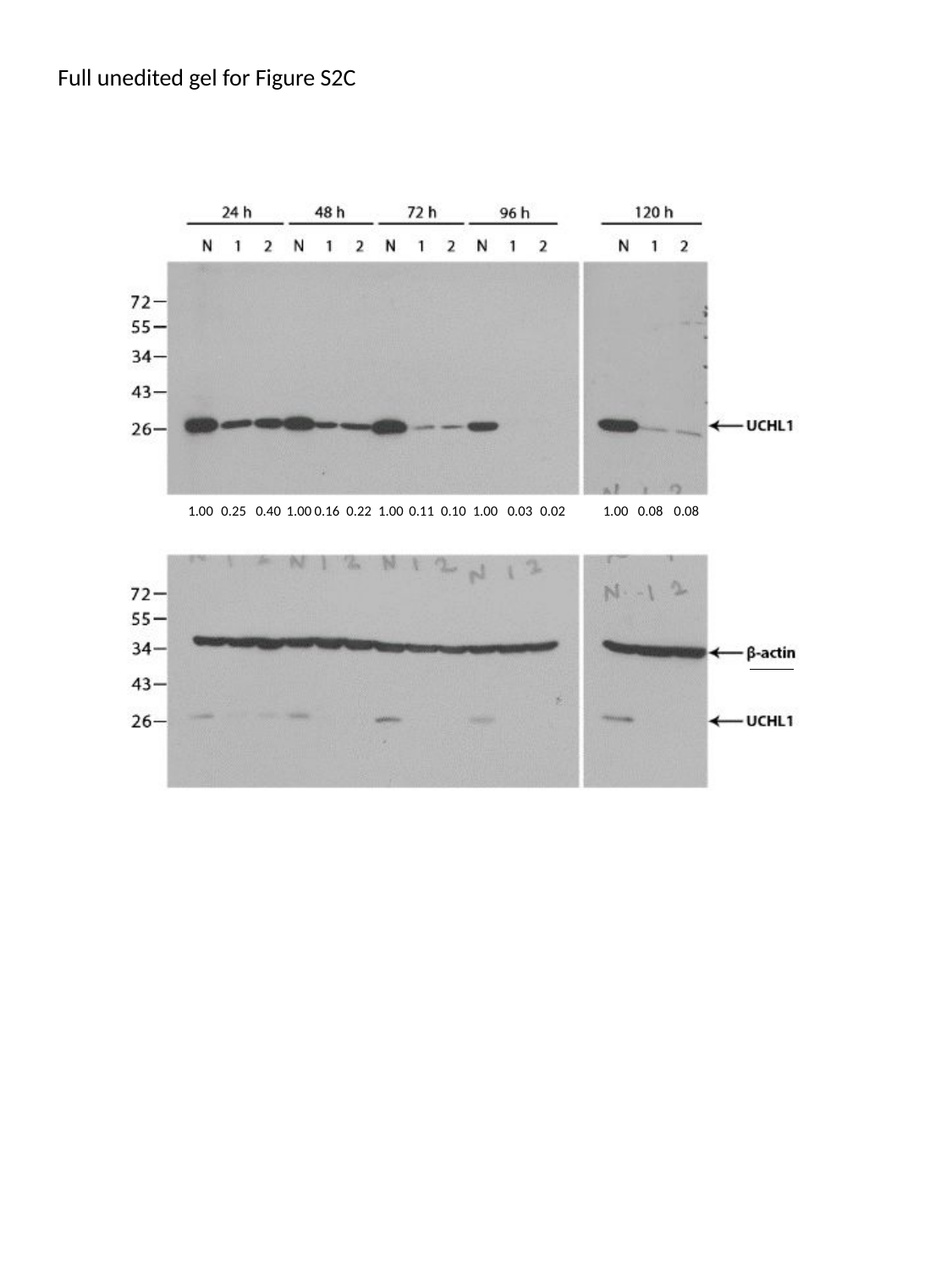

Full unedited gel for Figure S2C
1.00
0.25
0.40
1.00
0.16
0.22
1.00
0.11
0.10
1.00
0.03
0.02
1.00
0.08
0.08

## Slide 9
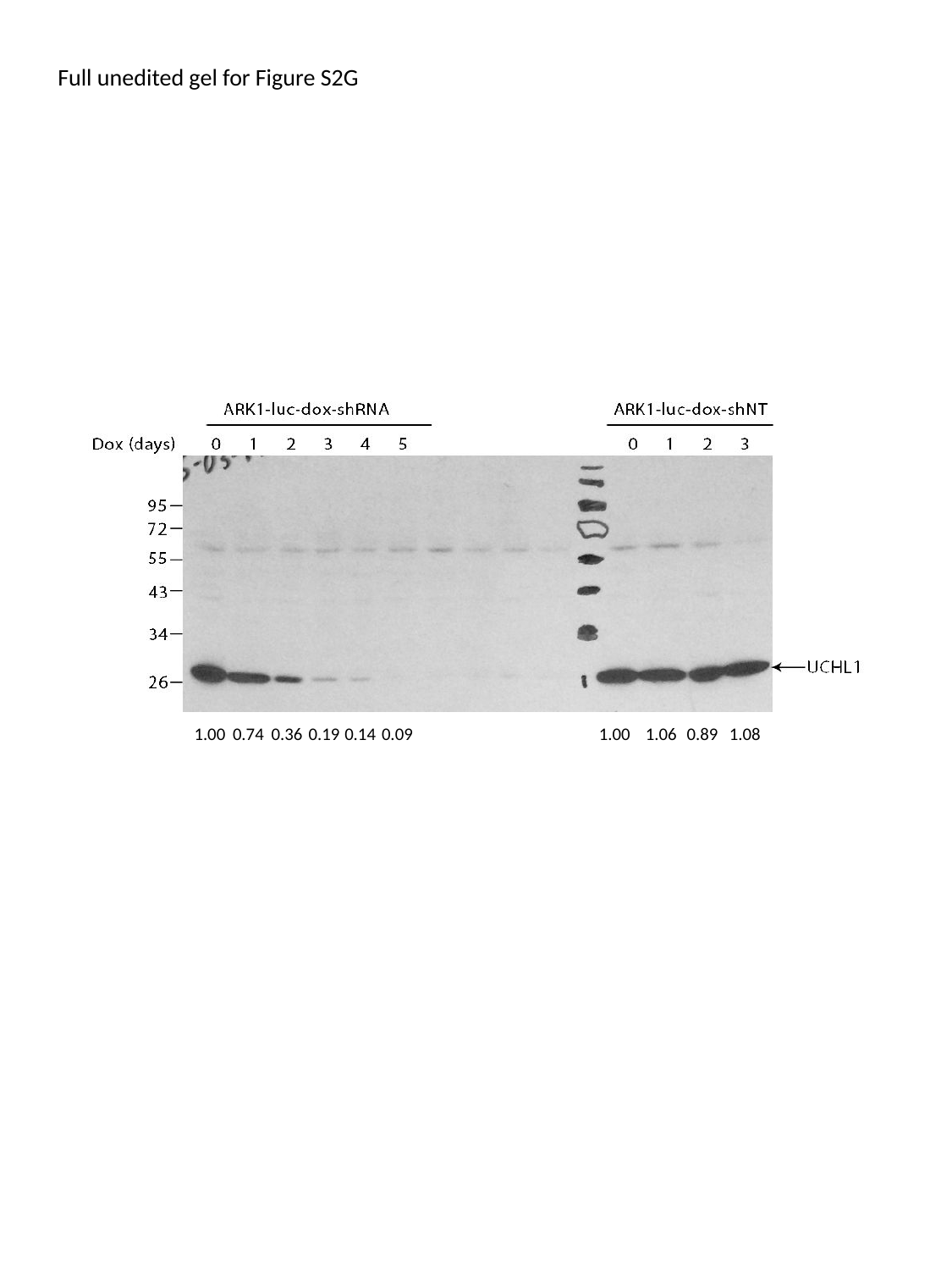

Full unedited gel for Figure S2G
1.00
0.74
0.36
0.19
0.14
0.09
1.00
1.06
0.89
1.08

## Slide 10
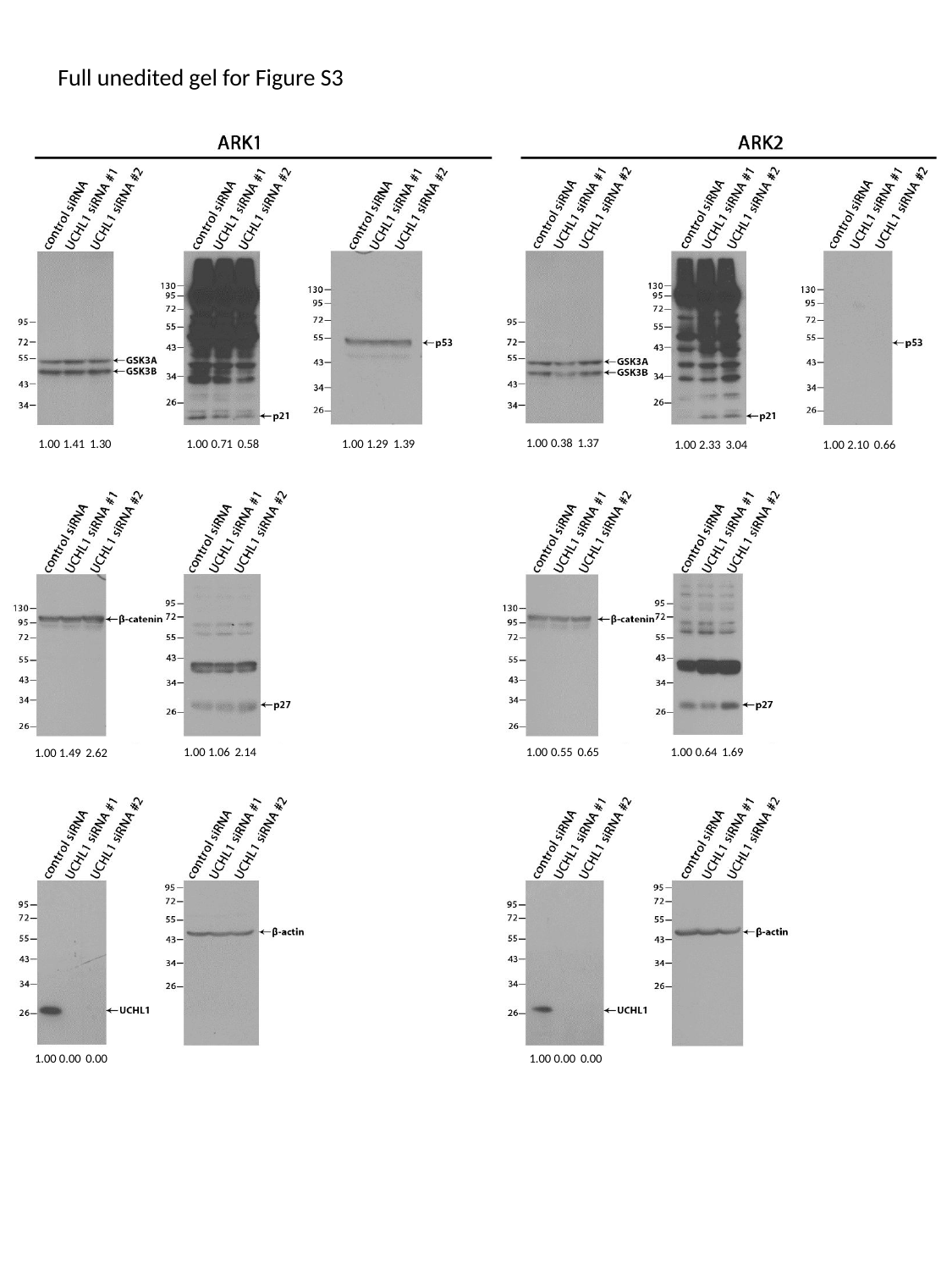

Full unedited gel for Figure S3
1.00
0.38
1.37
1.00
1.41
1.30
1.00
0.71
0.58
1.00
1.29
1.39
1.00
2.33
3.04
1.00
2.10
0.66
1.00
0.55
0.65
1.00
0.64
1.69
1.00
1.06
2.14
1.00
1.49
2.62
1.00
0.00
0.00
1.00
0.00
0.00
